# Photolithographic fabrication of high-resolution Micro-QLEDs towards color-conversion microdisplay

**DOI:** 10.1038/s41377-025-02000-y

**Published:** 2025-10-20

**Authors:** Yuyu Jing, Mingyu Yao, Min Yang, Menglin Li, He Ding, Gaoling Yang, Rongjian Zhang, Dengbao Han, Huan Liu, Haizheng Zhong

**Affiliations:** 1 MIIT Key Laboratory for Low-Dimensional Quantum Structure and Devices, School of Materials Science and Engineering, Beijing Institute of Technology, Beijing, 100081 China; 2 Beijing Engineering Research Center of Mixed Reality and Advanced Display, School of Optics and Photonics, Beijing Institute of Technology, Beijing, 100081 China; 3 Hefei Innovation Research Institute of Beihang University, Hefei, 230012 China; 4 Key Laboratory of Bio-Inspired Smart Interfacial Science and Technology of the Ministry of Education, School of Chemistry, Beihang University, Beijing, 100191 China

**Keywords:** Displays, Quantum dots

## Abstract

Microdisplay panels are critical components for metaverse technology. Aiming to achieve high-resolution and full-color microdisplay, we report the photolithographic fabrication of color-converted Micro-quantum dot light emitting diodes (QLED) panel by combining blue Micro-QLED electroluminescence (EL) device and red-green quantum dot color converter (QDCC). Pre-patterned templates were firstly photolithographically fabricated and then applied as substrate to fabricate patterned blue Micro-QLED device, achieving an ultra-high pixel resolution up to 6350 pixels per inch (pixel size ranging from 20 μm × 20 μm to 2 μm × 2 μm). Notably, the patterned blue devices achieve a peak external quantum efficiency (EQE) of 7.8% and a maximum brightness of 39,472 cd m^−2^. The patterned red devices achieve a peak EQE of 18% and a maximum brightness of 103,022 cd m^−2^. By integrating a dual-color red and green QDCC arrays on the top of the blue Micro-QLED, a prototype full-color Micro-QLED panel was fabricated, achieving a resolution up to 1184 pixels per inch with a peak EQE 4.8%, and a maximum brightness of 10 065 cd m^−2^. The photolithographic fabrication of color-converted Micro-QLED provides an easy-operated method for achieving cost-effective microdisplay panels.

## Abstract (web summary)

Full-color and high-resolution micro-quantum dot light emitting diodes (Micro-QLED) panel is fabricated by combining blue Micro-QLED electroluminescence (EL) device and red-green quantum dot color converter (QDCC).

## Graphical abstract

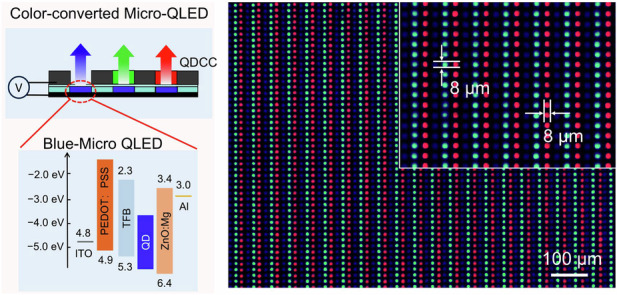

## Introduction

Metaverse is a platform to connect digital and physical realities, enabled by immersive technology of augmented reality (AR) and virtual reality (VR)^1–5^. As crucial components of AR and VR technology, microdisplay panels have attracted a great number of attentions from both academic and industrial researchers^6–13^. To date, there are a few of methodologies to achieve microdisplay panels, including liquid crystal on silicon (LCos)^14–17^, organic light emitting diode on silicon (OLEDos)^18–20^, and micro light emitting diode (Micro-LED)^21–26^. However, there are still great challenges to meet the requirements of high resolution and high brightness of AR technology, due to the brightness limitation of LCos and OLEDos and the full color limitation of Micro-LED. Quantum dot light-emitting diodes (QLED) are emerging as an alternative microdisplay technology due to their high brightness and solution-processed ability^27–34^. Therefore, it is of great importance to develop high-resolution patterning processing for fabricating high-resolution Micro-QLED, especially the full color panels^35,36^.

In the previous works, the fabrication of Micro-QLEDs strongly relies on the patterning of quantum dots (QDs), motivating the developments of inkjet printing^37–40^, transfer printing^41–43^, and direct photolithography of QDs^44–50^. After a decade of efforts, great successes have been made in patterning process of QDs, resulting in the fabrication of high resolution and full color QDs patterns^51,52^. To our knowledge, only a few works have evaluated electroluminescence (EL) devices of patterned QDs, due to the challenges of processing compatibility and current leakage between pixels with QLED fabrication process^37,41,42,53–59^. In this work, we develop a non-destructive and easy fabrication process for fabricating high-resolution Micro-QLEDs using pre-patterned photolithography templates, thereby enabling high-resolution blue and red Micro-QLEDs with a resolution up to 6350 pixels per inch (ppi) (pixel size ranging from 20 μm × 20 μm to 2 μm × 2 μm). Notably, the patterned blue devices achieve a peak EQE of 7.8% and a maximum brightness of 39 472 cd m^−2^, which is the highest resolution and EQE value in reported high-resolution QLEDs (Supplementary Table S1). The patterned red devices also achieve excellent performance with a peak EQE of 18% and a maximum brightness of 103,022 cd m^−2^. By integrating the dual-color (red/green) quantum dot color converter (QDCC) layer on a blue Micro-QLED panel, we further demonstrate full-color color-converted Micro-QLED displays achieving 1184 ppi resolution with a maximum brightness of 10 065 cd m^−2^ and a peak EQE of 4.8%, representing one of state-of-the-art performances in full-color Micro-QLED displays (Supplementary Table S2).

## Results

### Fabrication of color-converted Micro-QLED

**Fig. 1:**
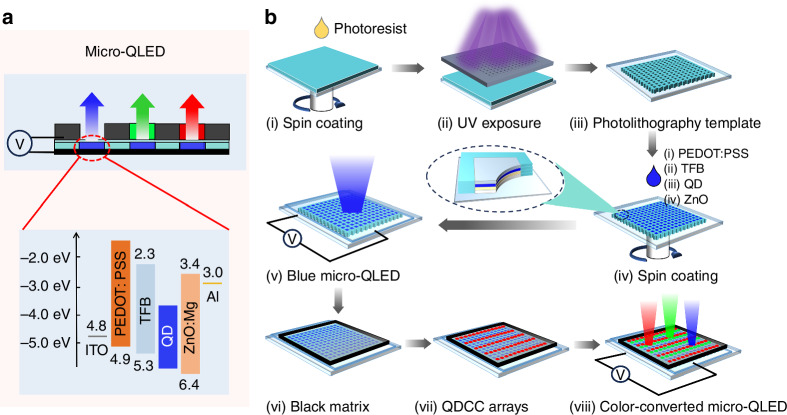
High-resolution full-color color-converted Micro-QLED using photolithography templates. **a** Schematic device structure of the color-converted Micro-QLED device. The inset shows the energy band diagram of patterned blue Micro-QLED. **b** Schematic diagram of the color-converted Micro-QLED device manufacturing process

Figure 1a shows the schematic structure of color-converted Micro-QLED that includes two components of blue Micro-QLED and dual-color (red/green) QDCC. The device structure of blue Micro-QLED is ITO/PEDOT: PSS/TFB/QDs/ZnMgO/Al. Figure 1b shows the fabrication process of color-converted Micro-QLED. First, the pre-patterned template was prepared by photolithography process including photoresist spin coating, UV exposure and developing. The functional layers of QLED were subsequently spin-coated on the pre-patterned templates, guiding the formation of micron-scale pixels, achieving a high-resolution blue-emitting Micro-QLED device. After that, we employed direct photolithography QDs photoresist to prepare red-green QDs arrays on a blue Micro-QLED panel, finally achieving a full-color color-converted Micro-QLED device with 8 μm × 8 μm sub-pixel.

**Fig. 2:**
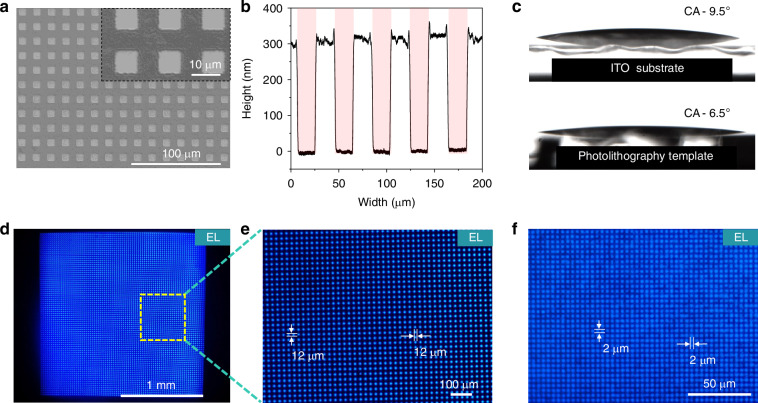
High-resolution blue Micro-QLEDs. **a** SEM images of photolithography template. Scale bar, 100 μm. **b** Height profile of the photolithography template pixels. **c** Water contact angle on the surface of the ITO substrate and photolithography template. **d** Electroluminescence (EL) image of blue patterned Micro-QLED device. EL active area is 1 mm^2^. Scale bar, 1 mm. Here **e** is a magnified optical microscope image of the marked square in (**d**). Scale bar, 100 μm. **f** Blue Micro-QLED with pixel size of 2 μm × 2 μm. Scale bar, 50 μm

Figure 2a and Supplementary Fig. S1 show the as-fabricated high-resolution photolithography template with the pixel size ranging from 20 μm × 20 μm to 2 μm × 2 μm. Cross-sectional analysis reveals that the photolithography templates have a depth of ~300 nm (Fig. 2b). As shown in Fig. 2c, the contact angle of ITO substrate with photolithography template is 6.5°, which is slightly less than that of bare ITO substrate of 9.5°. The reduced contact angle can be explained by the enhanced hydrophilicity due to the increased surface roughness of photolithography templates^60^. Figure 2d–f shows the as-fabricated 12 μm × 12 μm and 2 μm × 2 μm blue Micro-QLED devices. The Micro-QLED devices can emit blue light with uniform distribution (Supplementary Fig. S2 and Supplementary Fig. S3). With the driving voltage increasing up to 4 V, the spatially isolated pixelated EL were observed continuously within individual pixels (Supplementary Fig. S4).

### Monochrome Micro-QLED performance and analysis

**Fig. 3:**
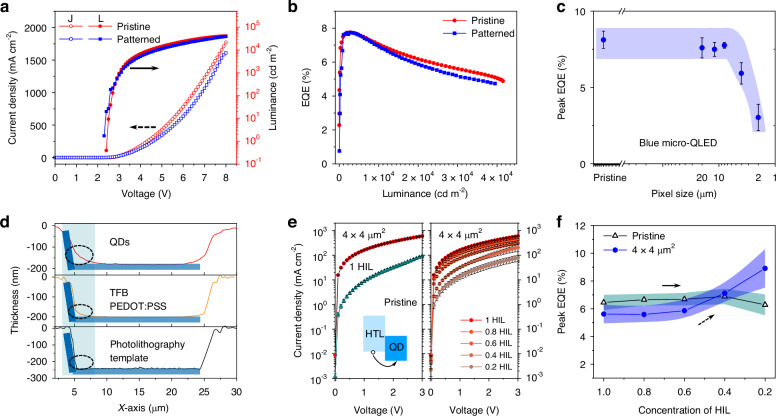
Device performance of blue Micro-QLEDs. **a** Current density (J)-voltage (V)-luminance (L) curves and (**b**) external quantum efficiency (EQE) versus luminance of blue Micro-QLED with a pixel of 12 μm × 12 μm and pristine QLED device. **c** pixel-sizes-dependent peak EQEs of multiple QLED devices (pristine blue QLED and blue Micro-QLED). Error bar represents the standard deviation of the data collected from >6 samples. **d** Height profiles of atomic force microscopy (AFM) images in the Micro-QLED manufacturing process. **e** J-V characteristics of hole-only device (HOD) using the pristine ITO substrate (initial HIL concentration, 1 HIL) and the 4 μm × 4 μm photolithography template (reduced HIL concentration). HOD structure: ITO/PEDOT: PSS/TFB/QDs/MoO_3_/Al. **f** Peak EQEs of blue Micro-QLED versus HIL concentration, using the ITO substrate and the 4 μm × 4 μm photolithography template

Figure 3a, b compare the device performance of blue pristine QLED and 12 μm × 12 μm Micro-QLED devices with the same device structure and fabrication process. These devices have similar current density (J)-voltage (V)- luminance (L) curves, external quantum efficiency (EQE) curves, and EL spectra (Supplementary Fig. S5), confirming the non-destructive feature of photolithography template assisted processing (PTA). This blue Micro-QLED device with a pixel of 12 μm × 12 μm has a peak EQE of 7.8% and a maximum luminance of 39,472 cd m^−2^. We further evaluate the influence of pixel size on blue Micro-QLED device performance. Figure 3c shows the evolution of average peak EQEs with pixel size decreasing from 20 μm × 20 μm to 2 μm × 2 μm. The average EQE of pristine devices is 8.1%. The average EQEs are 7.8%, 5% and 3% for the devices with pixel size of 20 μm × 20 μm, 4 μm × 4 μm, and 2 μm × 2 μm respectively. To illustrate the observed EQEs decreasing in Micro-QLEDs with small pixel sizes, we evaluate the influence of pixel size on red Micro-QLED device performance. As-fabricated patterned red Micro-QLED devices show uniform distribution with different pixel sizes from 12 μm × 12 μm to 2 μm × 2 μm (Supplementary Fig. S6–8). As shown in Supplementary Fig. S9 a–c, the patterned red Micro-QLED (12 μm × 12 μm) device shows a peak EQE of 18% and a maximum brightness of 103,022 cd m^−2^, similar with the pristine QLED device performance. As shown in Supplementary Fig. S9d, the red emissive pristine device and patterned devices also show similar device performance when the pixel sizes are larger than 8 μm × 8 μm. Meanwhile, similar EQEs decreasing phenomenon was also observed in the red Micro-QLEDs with small pixel sizes of 4 μm × 4 μm, and 2 μm × 2 μm.

To investigate the influence of pre-patterned substrates on device performance, we characterize the surface morphology of each functional layer of QLED device. Figure 3d and Supplementary Fig. S10 show the height profiles of atomic force microscopy (AFM) images of ITO, ITO/HIL/HTL, ITO/HIL/HTL/QDs. Thickness gradient increasing can be observed at the edges of the bank for all functional layers, due to the formation of a pile-up on the bank edges (pixel size of 20 μm × 20 μm). With the pixel size decreasing to 4 μm × 4 μm, the formation of a pile-up on the bank edges leads to the thickness increase of the functional layer in the entire pixel areas (Supplementary Fig. S11). The formation of the pile-up has been further demonstrated by AFM characterization on 4 μm × 4 μm device (Supplementary Fig. 12), corresponding to the EQEs decreasing. This phenomenon is attributed to the superior wettability of the side walls and bottom of photolithography template, which has also been observed in inkjet printing QLED processing^38,61,62^.

To further illustrate the EQEs decreasing in Micro-QLEDs with small pixel sizes, we investigate the influence of thickness on the device performance. We first compared the J-V characteristics of hole-only devices (HODs) for pristine and 4 μm × 4 μm devices. As shown in Fig. 3e, the current density (161.1 mA cm^−2^ at 1 V, and 594.1 mA cm^−2^ at 3 V) of 4 μm × 4 μm devices are almost 20 and 6 times than that (8.5 mA cm^−2^ at 1 V, and 92.3 mA cm^−2^ at 3 V) of pristine devices, suggesting imbalanced hole carrier injection in 4 μm × 4 μm devices. Therefore, the decrease of EQE can be attributed to increased current leakage paths and imbalanced hole injection. By reducing HIL solution concentration from 100% to 20% in 4 μm × 4 μm devices (Fig. 3e right), the HOD device can be greatly optimized, resulting in a current density decreasing from 594.1 mA cm^−2^ at 3 V to 78.2 mA cm^−2^ at 3 V, close to the values of pristine HODs (Supplementary Fig. S13 and Supplementary Fig. S14). The optimization of HIL layer can also induce balanced carrier injection, boosting peak EQEs from 5.5% to 9.0% for blue Micro-QLED with a pixel size of 4 μm × 4 μm (Fig. 3f) and 7.2% to 13.7% for red Micro-QLED with a pixel size of 4 μm × 4 μm (Supplementary Figs. S15–18).

### Full-color color-converted Micro-QLED performance

**Fig. 4:**
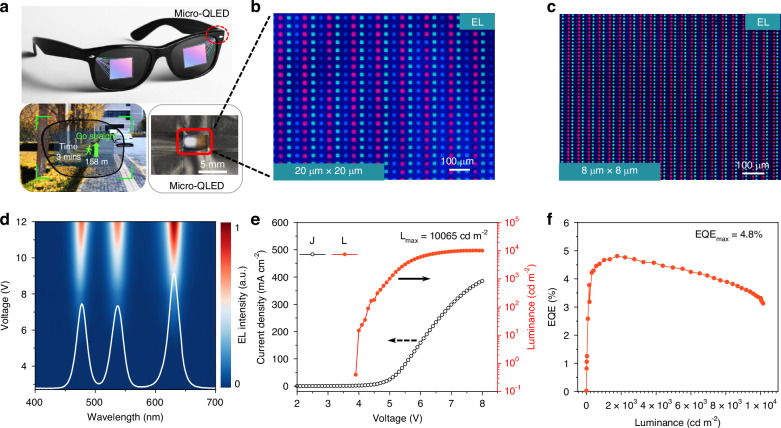
Full-color color-converted Micro-QLED device. **a** Schematic diagram of the potential application in AR display. EL image of full-color Micro QLED (right bottom). EL microscope images of color-converted Micro-QLED devices with the subpixel size of 20 μm × 20 μm (**b**) and 8 μm × 8 μm **c**. Scale bar, 100 μm. **d** Two-dimensional map of EL spectra of color-converted Micro-QLED device under various driving voltage from 4 V to 12 V. **e** J- V- L curves and **f**, EQE curves versus luminance of color-converted Micro-QLED with QDCC of 8 μm × 8 μm

To demonstrate potential application in AR technology, we fabricate a high-resolution full-color color-converted Micro-QLED panel by combining blue Micro-QLED device and dual-color red-green QDCC. As-fabricated full-color color-converted Micro-QLED panel was shown in the inset of Fig. 4a (right bottom). The schematic diagram of full-color Micro-QLED pixel is shown in Supplementary Fig. S19. The dual-color red and green QDCC was fabricated through direct photolithography of a QDs photoresist (QDPR) (Supplementary Fig. S20). The details of fabrication process are described in Experimental Section. The QDPR films with the thickness of 5 μm show blue light absorbance efficiency of ~96% and ~94% for red QDPR and green QDPR respectively. The photoconversion efficiency (PCE) are ~56% and ~51% for red QDPR and green QDPR (Supplementary Fig. S21). We fabricate the full-color Micro-QLED using green QDPR with thickness of 5.2 μm and red QDPR with thickness of 3.8 μm.

Figure 4b, c show the microscopic images of the red, green, and blue EL emission from color-converted Micro-QLED. The pixel resolutions of the panels are 473 ppi and 1184 ppi for sub-pixel 20 μm × 20 μm and 8 μm × 8 μm respectively. As shown in Fig. 4d, the panel can realize pure red, green, and blue EL emission with emission peaks at 476 nm, 537 nm, and 630 nm and full width at half maximum (FWHM) of 21 nm, 22 nm, and 21 nm respectively. The EL emission spectra of full-color panel under various driving voltage from 4 V to 12 V are shown in Fig. 4d. The CIE coordinate of the full-screen white light for the operated panel is (0.3515, 0.3893) with a coverage ratio 95.8% for the NTSC color gamut and a coverage ratio of 91.1% for the DCI-P3 color gamut (Supplementary Fig. S22). Figure 4e, f shows the J-V-L curves and calculated EQEs of the as-fabricated panel (statistical results from the full pixelated panel). All values are measured under full-screen white emission during operation. The panels achieve a peak brightness of 10065 cd m^−2^ and a peak EQE of 4.8% for QDCC of 8 μm × 8 μm and a peak brightness of 9235 cd m^−2^ and a peak EQE of 5.2% for QDCC of 20 μm × 20 μm (Supplementary Fig. S23). It is noticed that the brightness of color-converted Micro-QLED is mainly determined by blue QLED panel. Considering the AR applications, the brightness and efficiency can be further improved by optimizing the martials and device structures of blue QLED^34,63,64^. In addition, the photostability issues of QDCC also needs to be addressed^65,66^.

Supplementary Table S1 and Supplementary Table S2 summarize the literature reports on monochrome and full color Micro-QLED devices. Regarding the performance of Micro-QLED, only a few works evaluated the EL performance of pixelated QLEDs (Supplementary Fig. S24), due to the challenges of processing compatibility with QLED fabrication process and current leakage between pixels. The photolithography template assisted processing physically isolates the HIL/HTL layers of each pixel, which is critical to reduce electrical crosstalk^20^. The photolithography template assisted processing in this work is compatible with QLED fabrication process, enabling a non-destructive patterning of QLED devices, and achieving high-resolution and highly-efficient QLED devices. Supplementary Fig. S25 presents a comparison between as-fabricated device and previously reported Micro-QLED devices. As a state-of-the-art of the developments of Micro-QLED panels, the photolithography template assisted processing greatly improves the resolution and EQEs of monochrome Micro-QLEDs devices. The Blue Micro-QLEDs can achieve EQE 9.0% at 3175 ppi, and EQE 4.3% at 6350 ppi. The Red Micro-QLEDs can achieve EQE 15.8% at 3175 ppi, and EQE 13.5% at 6350 ppi. Compared to reported RGB Micro-QLED devices, the full-color color-converted Micro-QLED show advantages of resolution and EQE. These advances provide a new pathway for fabricating high resolution and high efficiency microdisplay panels compatible with AR/VR applications.

## Discussion

In summary, we have successfully demonstrated full-color color-converted Micro-QLED panel by combining blue Micro-QLED EL device and red-green QDCC array. The blue Micro-QLED device was fabricated by a non-destructive and easy fabrication process using photolithography pre-patterned templates, achieving a resolution up to 6350 pixels per inch (pixel size ranging from 20 μm × 20 μm to 2 μm × 2 μm). The patterned blue devices achieve a peak EQE of 7.8% and a maximum brightness of 39,472 cd m^−2^, which is one of the best device performances in high-resolution QLEDs. The patterned red devices also achieve high performance with a peak EQE of 18% and a maximum brightness of 103,022 cd m^−2^. The designed photolithography pre-patterned template can effectively address current leakage between pixels, achieving spatially isolated pixelated EL within individual pixels and similar EQEs with pristine QLED devices. The as-fabricated full-color Micro-QLED panel demonstrates an outstanding performance with 1184 ppi resolution, 4.8% peak EQE, and 10,065 cd m^−2^ maximum brightness, representing one of state-of-the-art Micro-QLED displays. The photolithography template assisted processing provide an easy-operated method to fabricate high resolution Micro-QLEDs, paving a new way of full-color microdisplay panels for AR and VR technology.

## Materials and Methods

### Experimental section

Chemicals: Zinc acetate dihydrate (Zn(OAc)₂·2H₂O), magnesium acetate tetrahydrate (Mg(OAc)₂·4H₂O), lithium hydroxide (LiOH), dimethyl sulfoxide (DMSO), and ethanol were purchased from Shanghai Aladdin Biochemical Technology Co., Ltd. The PEDOT:PSS 4083 aqueous solution (13 mg mL^−1^) was purchased from Xi’an Polymer Light Technology. Poly[(9,9-dioctylfluorenyl-2,7-diyl)-co-(4,4′-(N-(4-s-butylphenyl))diphenylamine)] (TFB) was purchased from Xi’an Polymer Light Technology at a concentration of 6 mg mL^−1^ in chlorobenzene for use. Quantum dots (QDs) with core-shell structures, including red (CdZnSe/CdZnS/ZnS) and blue (CdZnSe/ZnS) were provided by TCL Inc. Photoresist EOC-260 and red/green QD photoresist were supplied by the Hefei Innovation Research Institute of Beihang University. Photoresist SUN-2210N was purchased from Suntific Materials (Weifang) Co., Ltd.

Synthesis of ZnMgO nanoparticle solution: First, 1.5 mmol of Zn(OAc)₂·2H₂O and 0.15 mmol of Mg(OAc)₂·4H₂O were dissolved in 15 mL of dimethyl sulfoxide (DMSO) to prepare a precursor solution. Separately, 2.5 mmol of LiOH was dissolved in 25 mL of absolute ethanol, followed by 20 min of ultrasonication to ensure complete dissolution. The ethanol solution of lithium hydroxide was then slowly added dropwise into the DMSO solution, which contained zinc acetate and magnesium acetate. The mixture was stirred at 50 °C for 2 h to facilitate the formation of ZnMgO nanoparticles. After the reaction, the resulting product was thoroughly washed with ethanol and ethyl acetate several times to remove impurities. The ZnMgO nanoparticles were then dispersed in ethanol.

Photolithography process for high-resolution photolithography template:

The high-resolution photolithography template was fabricated using EOC-260 photoresist through standard lithography processing as follows:

i. Substrate Cleaning: Glass substrates underwent ultrasonic cleaning with deionized water, ethanol, and acetone (15 min each), followed by oxygen plasma treatment (100 W, 5 min).
ii. Photoresist Spin-Coating: EOC-260 photoresist was deposited via a three-stage spin coating process of 1000 rpm/s for 5 s, 3000 rpm/s for 5 s, and 7000 rpm/s for 40 s
iii. Soft Bake: Substrates were thermally baked at 80 °C for 5 min on a hotplate.
iv. UV Patterning: i-line lithography (365 nm) was used with 2-second exposure at 50–100 mJ cm-2 dose.
v. Development: Pattern development in propylene glycol monomethyl ether acetate (PGMEA) for ultrasonic cleaning 60 s to remove unexposed resist.
vi. Hard Bake: bake at 230 °C for 30 min to enhance structural integrity and thermal stability.

This process can produce photolithography templates with 2-micron resolution.

Micro-QLED device fabrication:

The QLED device structure is: ITO/PEDOT: PSS/TFB/QDs/ZnMgO/Al. Fabrication process initiates with oxygen plasma treatment (100 W, 5 min) of pre-patterned photolithography template on ITO substrates. A PEDOT: PSS layer was spin-coated (3500 rpm, 45 s) and thermally annealed (150 °C, 30 min) under ambient conditions. Subsequent processing was operated in an N₂-filled glovebox ( < 1 ppm O₂/H₂O).

The TFB layer (6 mg mL^−1^ in chlorobenzene) was deposited via spin-coating (2000 rpm, 30 s) followed by thermal annealing (150 °C, 30 min). Quantum dot (20 mg mL^−1^ in octane) was deposited via spin-coating (2000 rpm, 30 s) followed by thermal annealing (80 °C, 10 min). ZnMgO nanoparticle (20 mg mL^−1^ in ethanol) layers were sequentially spin-coated (2000 rpm, 30 s) followed by thermal annealing (90 °C, 30 min). Final Al electrodes were thermally evaporated (Kurt J. Lesker Mini Spectros, 2 × 10⁻⁷ Torr) through shadow masks. Pristine QLEDs were fabricated using standard ITO substrates. Patterned Micro-QLEDs were fabricated using pre-patterned templates prepared via the described process.

For hole-only devices (PEDOT: PSS/TFB/QDs/MoO_3_/Al), 8 nm MoO_3_ was thermally evaporated under identical vacuum conditions. All lithography templates were stored in N₂-filled glovebox prior to processing.

Photolithography process for dual-color red and green quantum dot color converter (QDCC):

The red/green QDCC array includes four functional components: a black barrier matrix, red QD photoresist patterns, green QD photoresist patterns, and blank pixels. The black barrier matrix was fabricated using SUN-2210N negative photoresist through the following lithography process:

i. Photoresist Deposition: Spin-coating at 1000 rpm/s for 20 s and 3000 rpm/s for 40 s to achieved uniform 3 μm-thick films.
ii. Soft Bake: Substrates were thermally baked at 80 °C for 7 min on a hotplate.
iii. UV Patterning: i-line (365 nm) exposure at 200 mJ cm^−2^ through chromium photomasks.
iv. Pattern Development: Immersion in TMAH-based alkaline developer (2.38 wt%) for 40 s to remove unexposed regions.

The red/green QDCC arrays were fabricated using red QDs photoresist and the green QDs photoresist. Using the pre-patterned black barrier matrix, the fabrication process are as follows:

i. Red QD Photoresist Patterning
  Spin-coating: at 500 rpm for 10 s and 3000 rpm for 50 s.
  Alignment and UV Patterning (365 nm): i-line (365 nm) exposure at 150 mJ cm^−2^ through chromium photomasks.
  Pattern Development: PGMEA immersion for 20 s to remove unexposed regions.
ii. Green QD Photoresist Patterning
  Spin-coating: at 500 rpm for 10 s and 3000 rpm for 50 s.
  Alignment and UV Patterning (365 nm): i-line (365 nm) exposure at 150 mJ cm^−2^ through chromium photomasks.
  Pattern Development: PGMEA immersion for 20 s to remove unexposed regions.

Finally, we obtained the red and green QDCC array. By integrating the QDCC array with the patterned blue QLED, we obtained the color-converted device.

Material characterization: Fluorescence microscopy images were taken using an optical microscope. QLED device characterization was performed at ambient temperature utilizing a J-V-L (current density-voltage-luminance) measurement system comprising: a photodetector (Thorlabs FDS1010), fiber-optic spectrometer (Avantes HSC-TEC), 6-inch integrating sphere, and source measurement unit (Keithley 2400). External quantum efficiency (EQE) values were calculated through the Lambertian emission model following established methods. Scanning electron microscope (SEM) images were scanned by a Regulus 8230 SEM system (Hitachi, Japan). Atomic force microscopy (AFM) images were measured by Dimension Fast Scan AFM system (Bruker, Germany).

## Supplementary information

Supplementary Information for Photolithographic fabrication of high-resolution Micro-QLEDs towards color-conversion microdisplay

## Data Availability

All the data supporting the findings of this study are available within this Article and its Supplementary Information. Any additional information can be obtained from the corresponding authors on reasonable request.

## References

[1] Xiong, J. H. et al. Augmented reality and virtual reality displays: emerging technologies and future perspectives. *Light Sci. Appl.* **10**, 216 (2021). 34697292 10.1038/s41377-021-00658-8 PMC8546092

[2] Ding, Y. Q. et al. Waveguide-based augmented reality displays: perspectives and challenges. *eLight* **3**, 24 (2023).

[3] Hsiang, E.-L. et al. AR/VR light engines: perspectives and challenges. *Adv. Opt. Photonics* **14**, 783–861 (2022).

[4] Qian, Y. Z. et al. Power consumption of light engines for emerging augmented reality glasses: perspectives and challenges. *Adv. Photonics* **7**, 034001 (2025).

[5] Artem, B. S. et al. Industrial applications of AR headsets: a review of the devices and experience. *Light Adv. Manuf.* **6**, 1–30 (2025).

[6] Behrman, K. & Kymissis, I. Micro light-emitting diodes. *Nat. Electron.* **5**, 564–573 (2022).

[7] Han, T.-H. et al. A roadmap for the commercialization of perovskite light emitters. *Nat. Rev. Mater.* **7**, 757–777 (2022).

[8] Dong, H. et al. Metal Halide Perovskite for next-generation optoelectronics: progresses and prospects. *eLight* **3**, 3 (2023).

[9] Liu, Z. J. et al. Micro-light-emitting diodes with quantum dots in display technology. *Light Sci. Appl.* **9**, 83 (2020). 32411368 10.1038/s41377-020-0268-1 PMC7214519

[10] Yuan, M. et al. Remote epitaxial crystalline perovskites for ultrahigh-resolution micro-LED displays. *Nat. Nanotechnol.* (2025). 10.1038/s41565-024-01841-9 39815067

[11] Li, J. H. et al. Efficient all-thermally evaporated perovskite light-emitting diodes for active-matrix displays. *Nat. Photonics* **17**, 435–441 (2023).

[12] Henry, W. & Percival, C. 55-2: Invited Paper: ILED displays: next generation display technology. SID Symp. *Dig. Tech. Pap.* **47**, 747–750 (2016).

[13] Ghosh, A. et al. 3.1: Invited Paper: OLED Micro-displays for VR/AR Applications. *SID Symp. Dig. Tech. Pap.* **50**, 26–27 (2019).

[14] Yin, K. et al. Advanced liquid crystal devices for augmented reality and virtual reality displays: principles and applications. *Light Sci. Appl.* **11**, 161 (2022). 35637183 10.1038/s41377-022-00851-3 PMC9151772

[15] Wu, Y.-H. et al. 5-2: Invited Paper: High dynamic range 2117-ppi LCD for VR displays. *SID Symp. Dig. Tech. Pap.* **54**, 36–39 (2023).

[16] Luo, Z. Y. et al. Ultracompact and high-efficiency liquid-crystal-on-silicon light engines for augmented reality glasses. *Opto-Electron. Adv.* **7**, 240039–240039 (2024).

[17] Song, J. X. et al. Polarized color filters using colloidal quantum rod nanocrystals for advanced high-performance displays. *Adv. Sci.* **12**, 2414316 (2025). 10.1002/advs.202414316 PMC12140335 40205696

[18] Lu, P. C. et al. 52-4: Highest PPI Micro-OLED display sustain for near-eye application. *SID Symp. Dig. Tech. Pap.* **50**, 725–726 (2019).

[19] Kang, C. -m & Lee, H. Recent progress of organic light-emitting diode microdisplays for augmented reality/virtual reality applications. *J. Inf. Disp.* **23**, 19–32 (2022).

[20] Kweon, H. et al. Microlithography of hole transport layers for high-resolution organic light-emitting diodes with reduced electrical crosstalk. *Nat. Electron.* **8**, 66–74 (2025).

[21] Park, J. et al. Electrically driven mid-submicrometre pixelation of InGaN micro-light-emitting diode displays for augmented-reality glasses. *Nat. Photonics* **15**, 449–455 (2021).

[22] Shin, J. et al. Vertical full-colour micro-LEDs via 2D materials-based layer transfer. *Nature* **614**, 81–87 (2023). 36725999 10.1038/s41586-022-05612-1

[23] Ayush, P. et al. Recent progress on micro-LEDs. *Light Adv. Manuf.* **4**, 519–542 (2024).

[24] Huang, J. H. et al. Monolithic integration of full-color microdisplay screen with sub-5 µm quantum-dot pixels. *Adv. Mater.* **36**, 2409025 (2024). 10.1002/adma.202409025 39267409

[25] Lin, C.-C. et al. The micro-LED roadmap: status quo and prospects. *J. Phys. Photonics* **5**, 042502 (2023).

[26] Velpugonda, J. L. et al. A universal high-resolution micro-patterning technique for solution-processed materials. *Light Adv. Manuf.* **6**, 1–8 (2025).

[27] Jang, E. & Jang, H. Review: quantum dot light-emitting diodes. *Chem. Rev.* **123**, 4663–4692 (2023). 36795794 10.1021/acs.chemrev.2c00695

[28] Shirasaki, Y. et al. Emergence of colloidal quantum-dot light-emitting technologies. *Nat. Photonics* **7**, 13–23 (2013).

[29] Liu, M. X. et al. Colloidal quantum dot electronics. *Nat. Electron.* **4**, 548–558 (2021).

[30] Sun, Q. J. et al. Bright, multicoloured light-emitting diodes based on quantum dots. *Nat. Photonics* **1**, 717–722 (2007).

[31] Dai, X. L. et al. Solution-processed, high-performance light-emitting diodes based on quantum dots. *Nature* **515**, 96–99 (2014). 25363773 10.1038/nature13829

[32] Zhang, M. et al. Ultrasmooth quantum dot micropatterns by a facile controllable liquid-transfer approach: low-cost fabrication of high-performance QLED. *J. Am. Chem. Soc.* **140**, 8690–8695 (2018). 29894177 10.1021/jacs.8b02948

[33] Zhang, W. J. et al. Stable and efficient pure blue quantum-dot LEDs enabled by inserting an anti-oxidation layer. *Nat. Commun.* **15**, 783 (2024). 38278797 10.1038/s41467-024-44894-z PMC10817946

[34] Wu, Q. Q. et al. Homogeneous ZnSeTeS quantum dots for efficient and stable pure-blue LEDs. *Nature* (2025). 10.1038/s41586-025-08645-4 40044864

[35] Li, D. P. et al. 56-1: Invited paper: quantum dot light-emitting diodes for micro display. *SID Symp. Dig. Tech. Pap.* **55**, 484–486 (2024).

[36] Lee, J. et al. Recent progress on quantum dot patterning technologies for commercialization of QD-LEDs: current status, future prospects, and exploratory approaches. *Small Methods* **8**, e2301224 (2024). 38193264 10.1002/smtd.202301224

[37] Kim, B. H. et al. High-resolution patterns of quantum dots formed by electrohydrodynamic jet printing for light-emitting diodes. *Nano Lett.* **15**, 969–973 (2015). 25584701 10.1021/nl503779e

[38] Roh, H. et al. Enhanced performance of pixelated quantum dot light-emitting diodes by inkjet printing of quantum dot–polymer composites. *Adv. Opt. Mater.* **9**, (2021).

[39] Haverinen, H. M. et al. Inkjet printing of light emitting quantum dots. *Appl. Phys. Lett.* **94**, 073108 (2009).

[40] Kang, C. B. et al. Inkjet printed patterned bank structure with encapsulated perovskite colour filters for modern display. *Nanoscale* **14**, 8060–8068 (2022). 35608246 10.1039/d2nr00849a

[41] Kim, T.-H. et al. Full-colour quantum dot displays fabricated by transfer printing. *Nat. Photonics* **5**, 176–182 (2011).

[42] Meng, T. T. et al. Ultrahigh-resolution quantum-dot light-emitting diodes. *Nat. Photonics* **16**, 297–303 (2022).

[43] Yoo, J. et al. Highly efficient printed quantum dot light-emitting diodes through ultrahigh-definition double-layer transfer printing. *Nat. Photonics* **18**, 1105–1112 (2024).

[44] Maeng, S. & Cho, H. Direct optical lithography: toward nondestructive patterning of nanocrystal emitters. *Accounts of Mater. Res.* (2025).

[45] Wang, Y. Y. et al. Direct optical lithography of functional inorganic nanomaterials. *Science* **357**, 385–388 (2017). 28751606 10.1126/science.aan2958

[46] Nie, Q. et al. Direct optical patterning of quantum dot light-emitting diodes based on ultrafast, low-energy, site-controlled click chemistry reaction. *Adv. Funct. Mater.* **35**, 2420829 (2025).

[47] Pan, J.-A. et al. Direct optical lithography of colloidal metal oxide nanomaterials for diffractive optical elements with 2π phase control. *J. Am. Chem. Soc.* **143**, 2372–2383 (2021). 33508190 10.1021/jacs.0c12447

[48] Fu, Z. et al. Direct photopatterning of colloidal quantum dots with electronically optimized diazirine cross-linkers. *J. Am. Chem. Soc.* **146**, 28895–28905 (2024). 39381921 10.1021/jacs.4c09209

[49] Seo, H. et al. Direct photolithography of colloidal InP-based quantum dots utilizing the photoligation method. *Chem. Mater.* **37**, 1424–1431 (2025).

[50] Wei, Y. et al. In situ light-initiated ligands cross-linking enables efficient all-solution-processed perovskite light-emitting diodes. *J. Phys. Chem. Lett.* **11**, 1154–1161 (2020). 31967835 10.1021/acs.jpclett.9b03775

[51] Zou, Y. T. et al. Recent progress on patterning strategies for perovskite light-emitting diodes toward a full-color display prototype. *Small Sci.* **1**, 2000050 (2021). 40213164 10.1002/smsc.202000050 PMC11935914

[52] Nam, T. W. et al. Ultrahigh-resolution quantum dot patterning for advanced optoelectronic devices. *Chem. Commun.* **59**, 2697–2710 (2023). 10.1039/d2cc05874j 36751869

[53] Li, Y. Z. et al. 80-1: Invited paper: developing AMQLED technology for display applications. *SID Symp. Dig. Tech. Pap.* **49**, 1076–1079 (2018).

[54] Mei, W. H. et al. High-resolution, full-color quantum dot light-emitting diode display fabricated via photolithography approach. *Nano Res.* **13**, 2485–2491 (2020).

[55] Zhao, J. Y. et al. Large-area patterning of full-color quantum dot arrays beyond 1000 pixels per inch by selective electrophoretic deposition. *Nat. Commun.* **12**, 4603 (2021). 34326332 10.1038/s41467-021-24931-x PMC8322170

[56] Yang K. Y. et al. High-resolution and high-performance full-color electroluminescent quantum dot light-emitting diodes. *Nano Energy* 110817 (2025).

[57] Yang, J. et al. Nondestructive photopatterning of heavy-metal-free quantum dots. *Adv. Mater.* **34**, e2205504 (2022). 35985813 10.1002/adma.202205504

[58] Ma, T. et al. One-step, mask-free, rapid laser writing fabrication of electroluminescent perovskite@oxide pixels for ultra-high PPI, efficient micro-QLEDs. *Adv. Funct. Mater.* **35**, 2413811 (2025).

[59] Wang, C. Y. et al. High-resolution and high-efficiency micro quantum-dot light-emitting diode arrays via conventional photolithography. *Nano Res.* (2025).

[60] Yao, M. et al. A review of membrane wettability for the treatment of saline water deploying membrane distillation. *Desalination* **479**, 114312 (2020).

[61] Lee, Y. & Jeong, Y.-C. Dewetting-assisted inkjet printing and optimized solvent mixture of Cd-free InP quantum dots for high-resolution pixel-pattern. *Flex. Print. Electron.* **9**, 025012 (2024).

[62] Gao, Y. Y. et al. Inkjet-printed, flexible full-color photoluminescence-type color filters for displays. *Adv. Eng. Mater.* **24**, 2101553 (2022).

[63] Long, Z. W. et al. A reactivity-controlled epitaxial growth strategy for synthesizing large nanocrystals. *Nat. Synth.* **2**, 296–304 (2023).

[64] Zhou, T. Y. et al. High-performance tandem quantum-dot light-emitting diodes based on bulk-heterojunction-like charge-generation layers. *Adv. Mater.* **36**, 2313888 (2024). 10.1002/adma.202313888 38488320

[65] Gao, Z. Y. et al. Quantum Dots Photoresist for Direct Photolithography Patterning. *Adv. Optical Mater.* **12**, 2401106 (2024).

[66] Zhang, P. P. et al. Direct in situ photolithography of perovskite quantum dots based on photocatalysis of lead bromide complexes. *Nat. Commun.* **13**, 6713 (2022). 36344550 10.1038/s41467-022-34453-9 PMC9640639

