# Supplementary material for: Photolithographic fabrication of high-resolution Micro-QLEDs towards color-conversion microdisplay
Source: Light Sci Appl. 2025 Oct 20;14:370. doi: 10.1038/s41377-025-02000-y (PMC12536041; doi:10.1038/s41377-025-02000-y)
Supplement: Supplementary file 1 — Supplementary Information for Photolithographic fabrication of high-resolution Micro-QLEDs towards color-conversion microdisplay [file 41377_2025_2000_MOESM1_ESM.docx]

Supplementary Information

**Photolithographic fabrication of high-resolution Micro-QLEDs towards color-conversion microdisplay**

Yuyu Jing ^1^, Mingyu Yao ^1^, Min Yang ^1^, Menglin Li^1^, He Ding^2^, Gaoling Yang^2^, Rongjian Zhang^3^, Dengbao Han ^3,4^, Huan Liu ^4^, Haizheng Zhong ^1^ *

^1^ MIIT Key Laboratory for Low-Dimensional Quantum Structure and Devices, School of Materials Science and Engineering, Beijing Institute of Technology, Beijing 100081, China

^2^ Beijing Engineering Research Center of Mixed Reality and Advanced Display, School of Optics and Photonics, Beijing Institute of Technology, Beijing, 100081, China

^3^ Hefei Innovation Research Institute of Beihang University, Hefei 230012, China

^4^ Key Laboratory of Bio-Inspired Smart Interfacial Science and Technology of the Ministry of Education, School of Chemistry, Beihang University, Beijing 100191, China

*Corresponding authors. E-mail addresses: hzzhong@bit.edu.cn

**Supplementary Table S1**. Performance comparison with reported patterned monochrome QLED devices.

| Method | Fabrication  method | Material | Color | PL pixel size / PPI | EL pixel size /PPI | EL pixel array peak  EQE | Ref: |
| --- | --- | --- | --- | --- | --- | --- | --- |
| Photolithography | Photolithographic template，photoresist | CdSe | R | N/A | 1588  3175  6350 | R 18.8 %  R 15.8%  R 13.7 % | This work |
| Photolithography | Photolithographic template，photoresist | CdSe | B | N/A | 1588  3175  6350 | B 8.1 %  B 9%  B 4.3% | This work |
| Photolithography | ligand  crosslinking, mask | CdSe, InP | R, G, B | 1270 | N/A | N/A | 1 |
| Photolithography | photoacid generator,  in situ ligand exchange，  mask | CdSe | R, G, B | Line 1.5 μm | N/A | N/A | 2 |
| Photolithography | ligand crosslinking,  mask | CdSe | R, G | ~5000 | N/A | N/A | 3 |
| Photolithography | ligand cleavage | CdSe，CdZnS | R, G, B | N/A | N/A | 5.6% | 4 |
| Photolithography | ligand  crosslinking,  mask | InP，CdSe，PbS | R, G, B | ~12700 | N/A | N/A | 5 |
| Photolithography | ligand  crosslinking,  mask | CdZnSe | R, G, B | Line ~3 μm | N/A | N/A | 6 |
| Photolithography | ligand  crosslinking, mask | CdSe, InP | R, G, B | 15875 | ~30 | N/A | 7 |
| Photolithography | ligand  crosslinking, mask | CsPbX_3_, FAPbX_3_ | R, G, B | ~1664 | ~400 | N/A | 8 |
| Photolithography | Photolithographic template，photoresist SU8 | CdSe | R,  G,  B | N/A | ~6000 | 16.5%, 20.1%,  12.7% | 9 |
| Inkjet printing | electrohydrodynamic printing | CdSe/CdZnSeS, CdSe/CdS/ZnS | R, G | ~465 | ~421 | R 2.6%  G 2.5% | 10 |
| Inkjet printing | electrohydrodynamic printing | CdSe | RG two color | 500 | 500 | N/A | 11 |
| Inkjet printing | electrohydrodynamic printing | CdSe | R | 306 | 306 | 0.55% | 12 |
| Transfer printing |  | CdSe/ZnS | R, G | 9072,  25400 | 9072 | R 14.72%，  G 7.6% | 13 |
| Screen printing |  | MAPbX_3_ | R, G, B | ~508 | ~550 | G<0.1% | 14 |
| Transfer printing |  | CsPbX_3_ | R, G | 2550 | 2550 | N/A | 15 |
| Transfer printing |  | CdSe | R, G, B | 2565, 20526 | N/A | N/A | 16 |
| Electrophoretic deposition | patterned three electrode substrates | CdSe | R, G, B | 252, 1093 | N/A | N/A | 17 |
| Electrostatic force-induced deposition | photoresist | CdSe, CsPbBr_3_ | R,  G | 1104,  3031 | N/A | R 15.6%，  G 3.9% | 18 |
| Laser method | Continuous wave laser | CsPbX_3_ | R | 10160 | 5000 | R 10.2%  G 15.8%  B 4.4% | 19 |
| Laser method | Femtosecond  laser | 2D perovskites | G | ~5000 | N/A | NA | 20 |
| Electrophoretic deposition | Orthogonal alternating electric field, photoresist | CdSe, CsPbBr_3_ | R, | 254,  507,  1037,  23090 | 254,  507,  1037, | 16.5%,  14.4%  11.7% | 21 |

**Supplementary Table S2**. Performance comparison with reported patterned full-color Micro-QLED devices.

| Method | Fabrication  method | Materials | Color | PL PPI | EL pixel size PPI | EL array  EQE max | Ref: |
| --- | --- | --- | --- | --- | --- | --- | --- |
| Photolithography | Photolithographic template，QD photoresist | CdSe | Full color | N/A | 1184 | 4.8% | This work |
| Transfer printing | TFT | CdSe，  4-inch demo | Full color | N/A | ~100 | N/A | ^22^ |
| Inkjet printing | TFT | 5- and 14-inch demo AMQLED | Full color | N/A | ~80 | N/A | ^23^ |
| Photolithography | Photoresist, lift-off, mask | CdSe | Full color | 500 | 500 | R 8.3 cd·A^-1^,  G 9.8 cd·A^-1^,  B 0.02 cd·A^-1^ | ^24^ |
| Photolithography | ligand  crosslinking | CdSe | Full-color | 1400 | N/A | N/A | 3 |
| Photolithography | ligand  crosslinking,  mask | InP | Full color | 3240 | N/A | N/A | 5 |
| Transfer printing |  | CsPbX_3_ | Full color | 847, 1693, 2550 | N/A | N/A | ^15^ |
| Transfer printing |  | CdSe | Full color | 2565 | N/A | N/A | ^16^ |
| Transfer printing |  | CdSe | Full color | ~60 | ~60 | 1.6% | ^25^ |
| Transfer printing |  | CdSe | Full color | 6350 | 6350 | 3.36% | ^26^ |
| Electrophoretic deposition | patterned three electrode substrates | CdSe | Full color | 1075 | N/A | N/A | ^17^ |
| Asymmetric wettability interface | Photoresist, direct laser writing | CdSe | Full color | ~1278 | N/A | 0.944% | ^27^ |


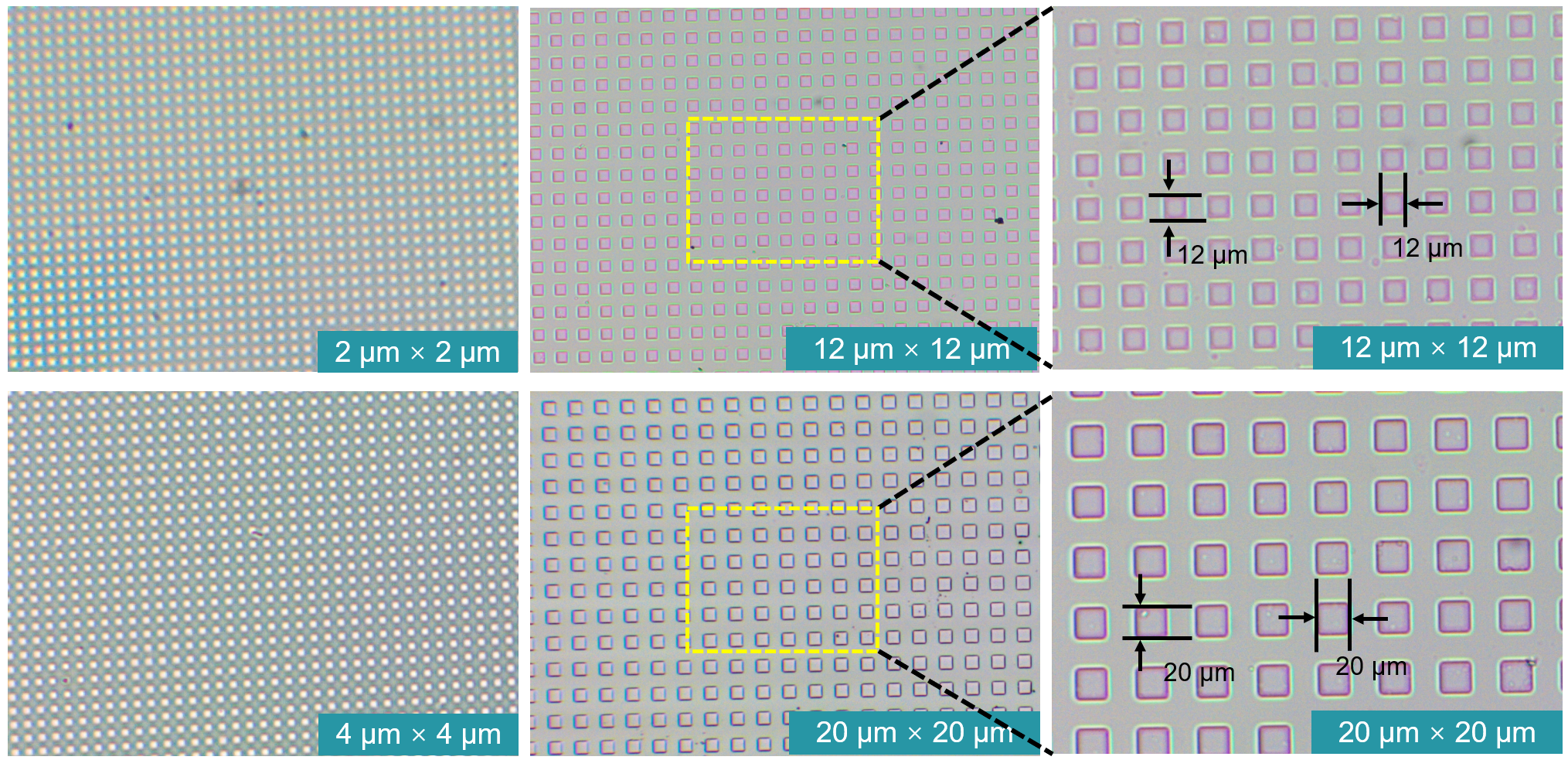


**Supplementary Fig. S1** Optical microscope image of photolithography template. Pixel sizes ranges from 2 μm × 2 μm to 20 μm × 20 μm.


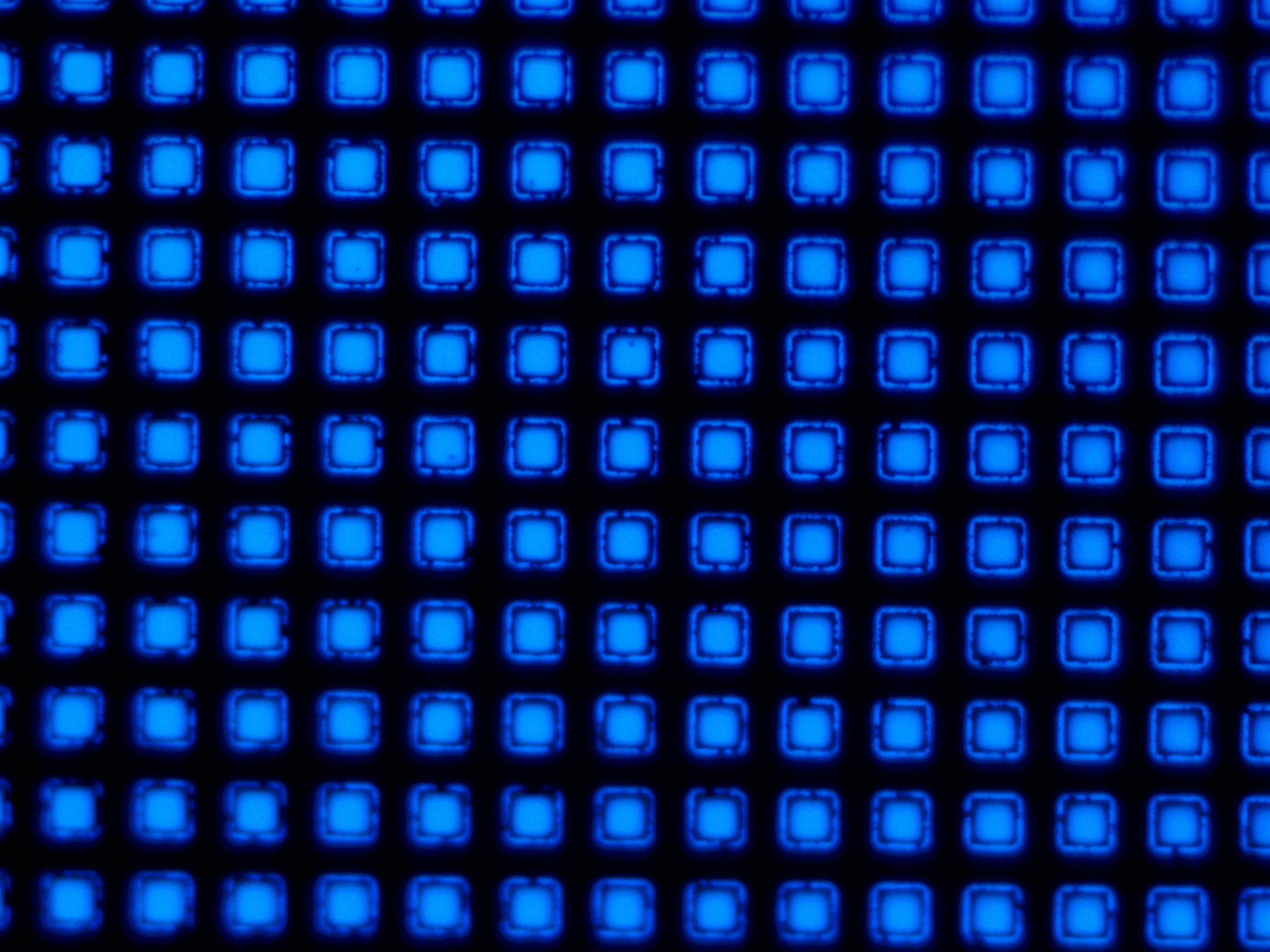


**Supplementary Fig. S2**. Electroluminescence (EL) optical microscope images of blue Micro-QLED device with pixel size of 20 μm × 20 μm.


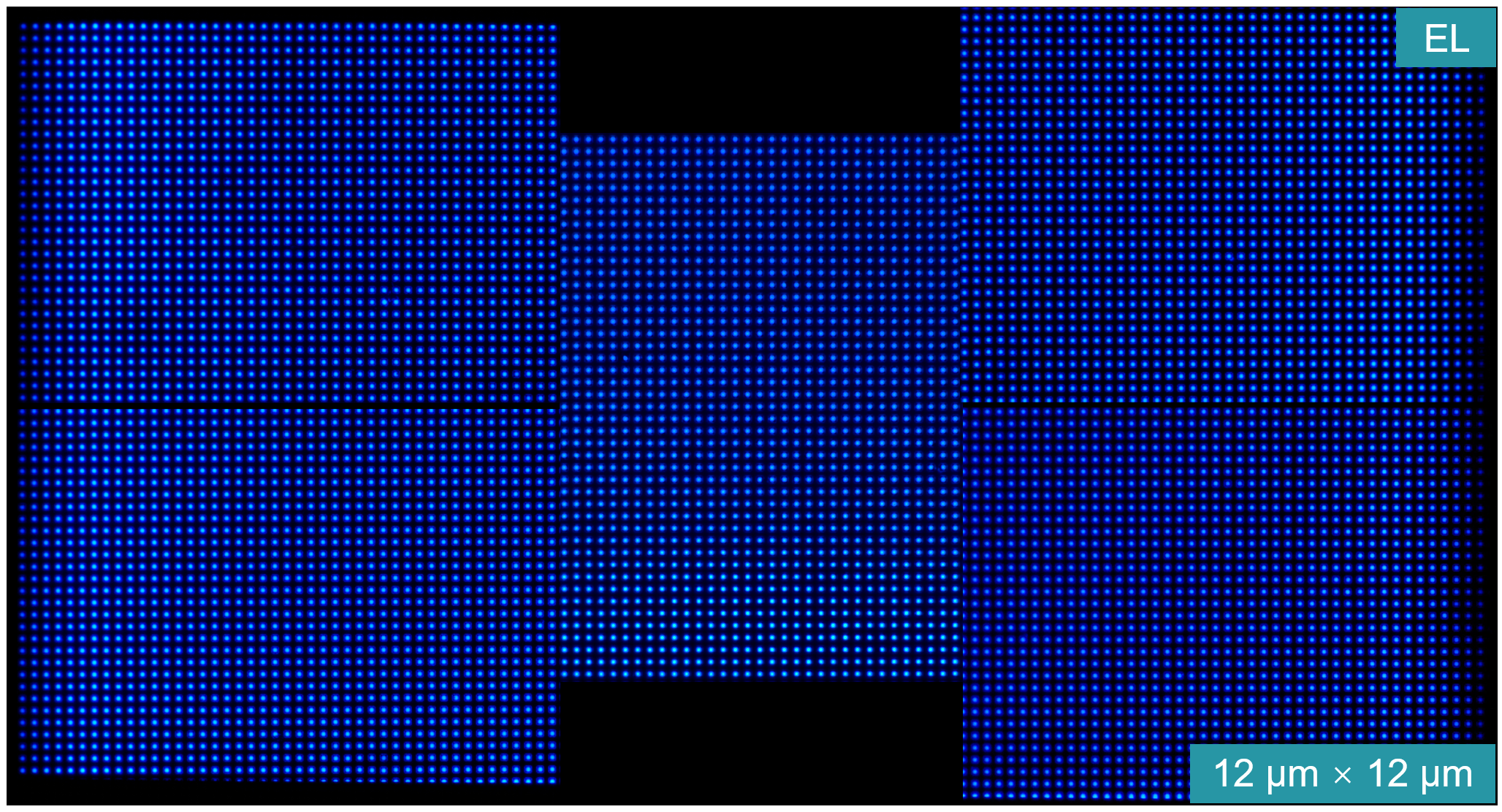


**Supplementary Fig. S3** EL optical microscope images of blue Micro-QLED device. EL active area is 4 mm^2^. Pixel size is 12 μm × 12 μm.


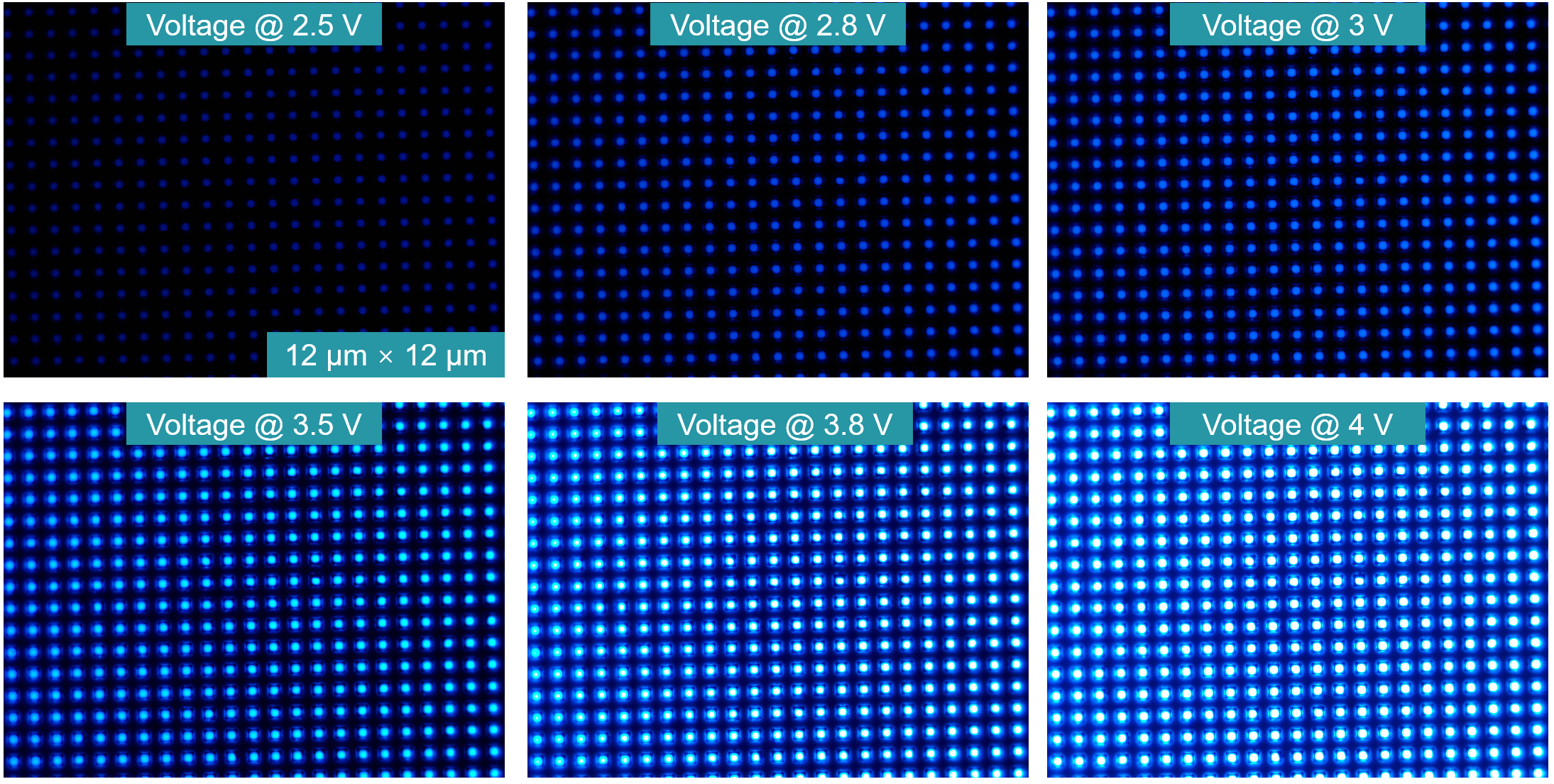


**Supplementary Fig. S4** EL optical microscope images of blue Micro-QLED device under a driving voltage of 2.5 – 4 V. Pixel size is 12 μm × 12 μm.


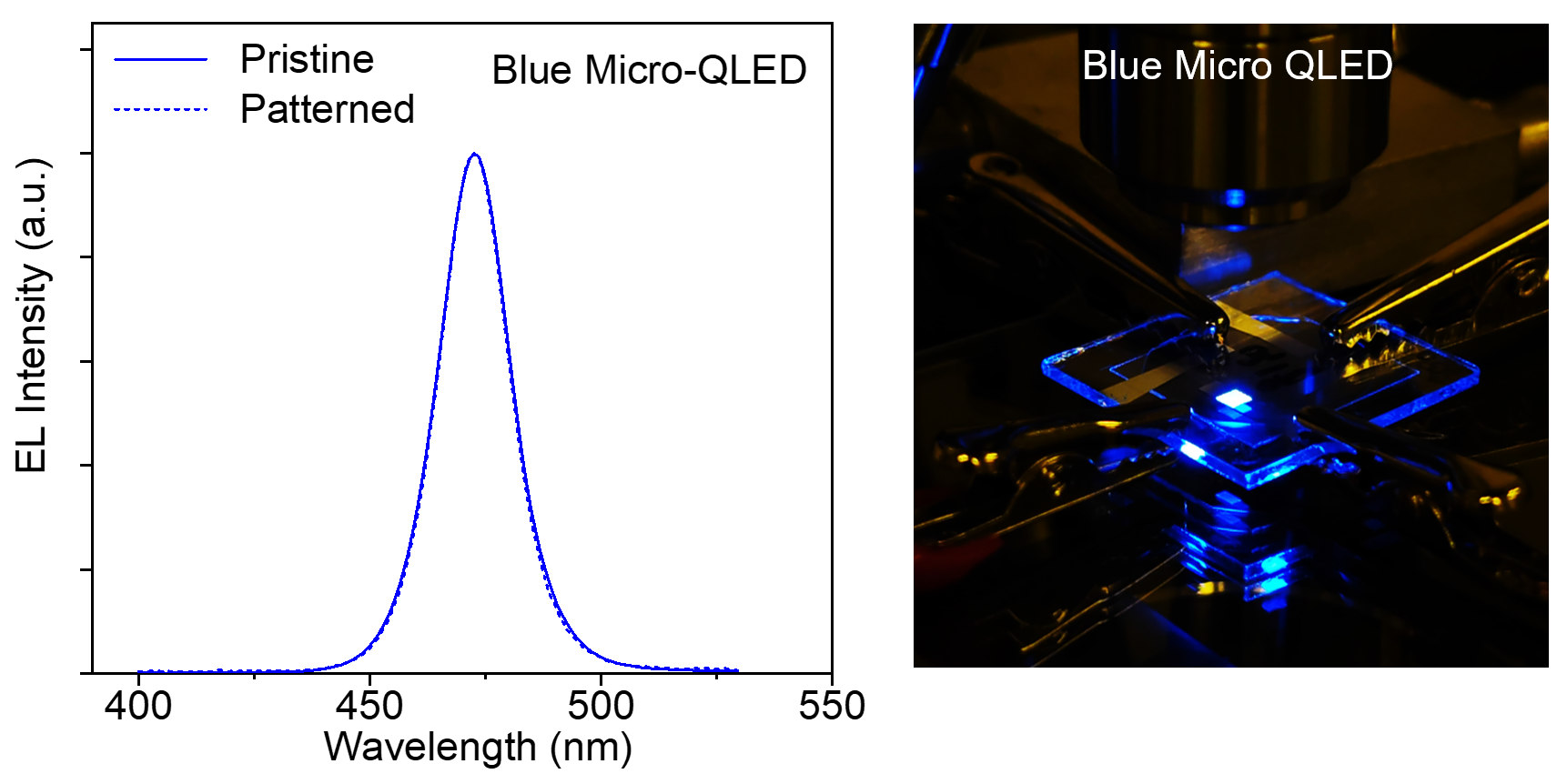


**Supplementary Fig. S5** EL spectra of blue Micro-QLED and pristine QLED devices and a photograph of the operating Micro-QLED


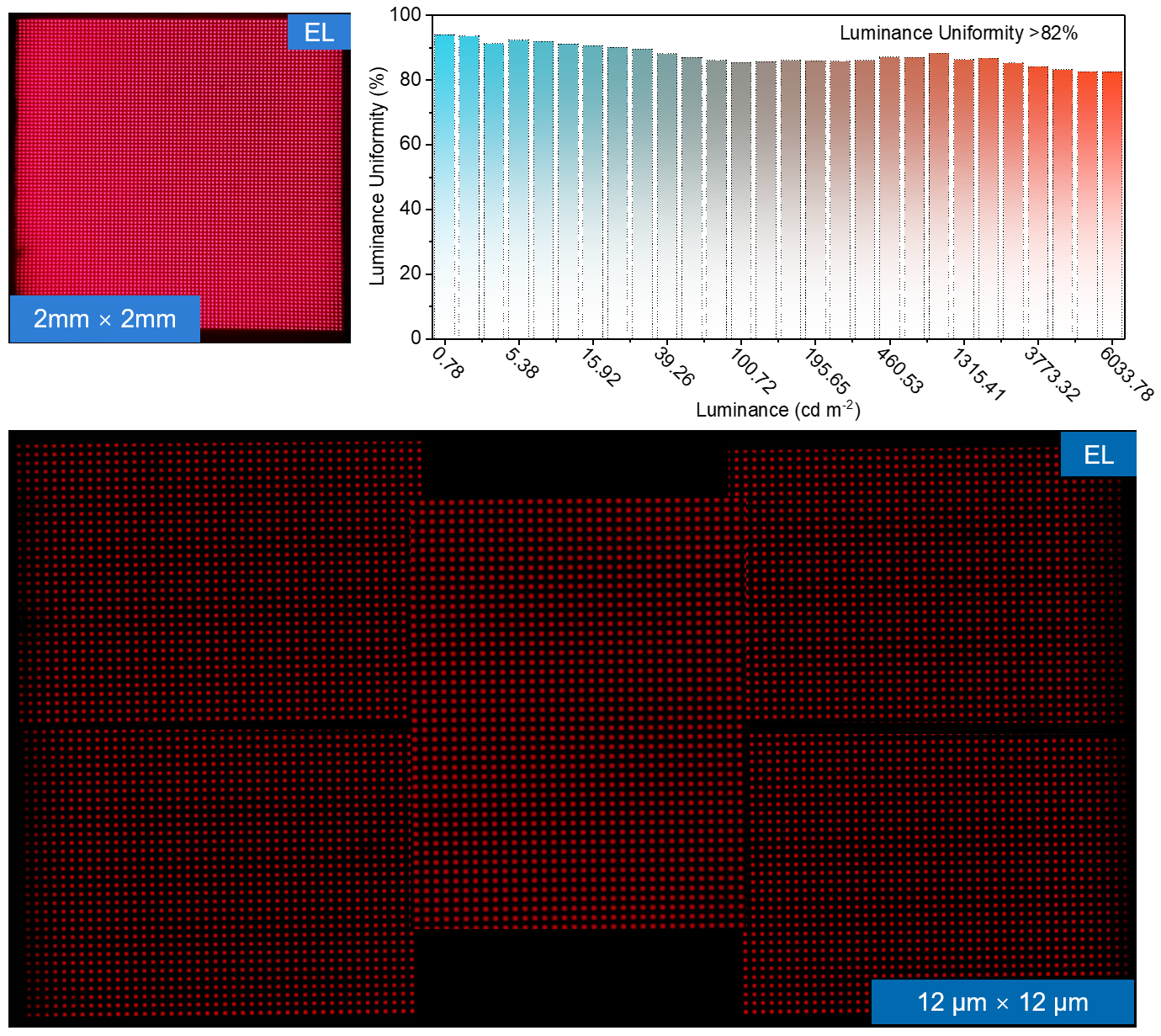


**Supplementary Fig. S6** EL image and optical microscope images of red Micro-QLED device. EL active area is 4 mm^2^. Pixel size is 12 μm × 12 μm. Luminance uniformity > 82%.


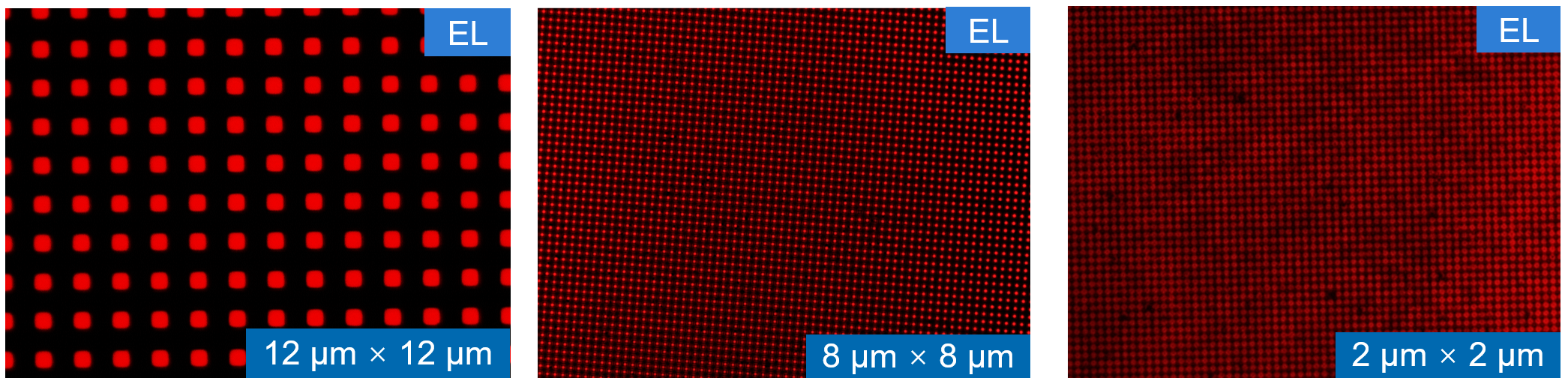


**Supplementary Fig. S7** EL optical microscope images of red Micro-QLED devices under the driving voltage. Pixel size is 12 μm × 12 μm, 8 μm × 8 μm, 2 μm × 2 μm.


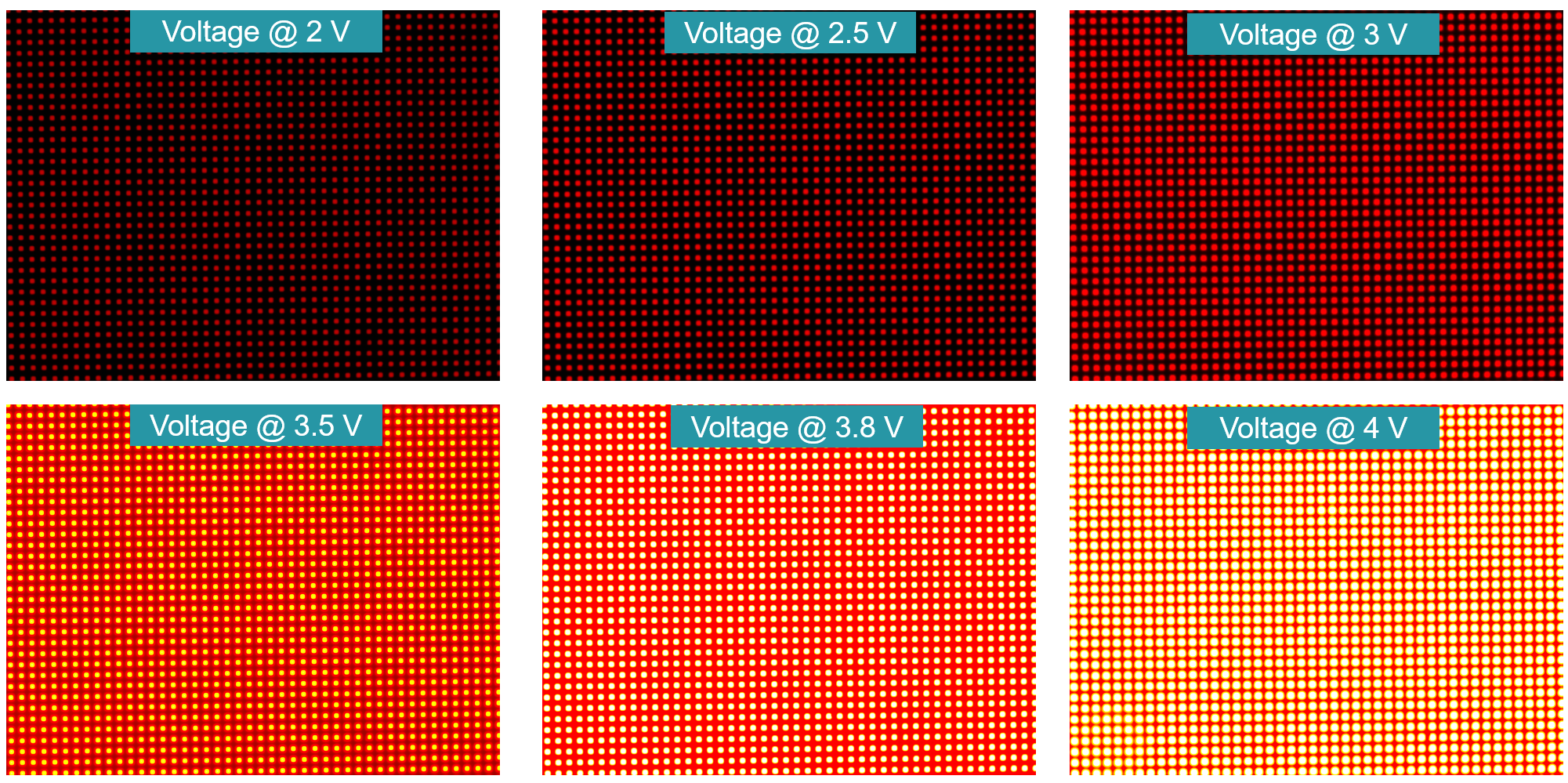


**Supplementary Fig. S8** EL optical microscope images of red Micro-QLED device under a driving voltage of 2 – 4 V. Pixel size is 12 μm × 12 μm.


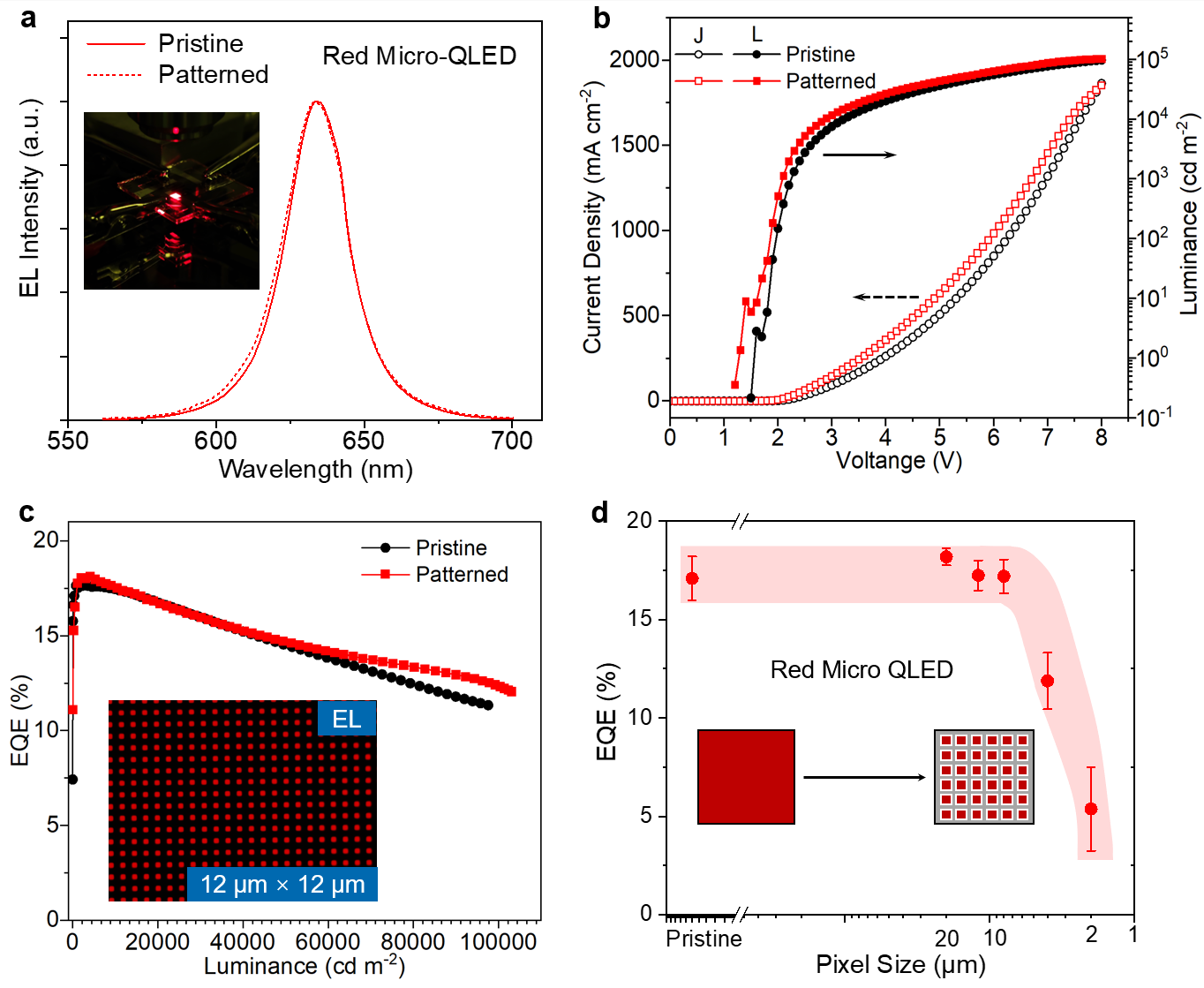


**Supplementary Fig. S9 a,** EL spectra of red Micro-QLED with a pixel of 12 μm × 12 μm and pristine QLED devices and a photograph of the operating Micro-QLED. **b**, J-V-L curves of red Micro-QLED and pristine QLED devices. **c**, EQE curves versus luminance of patterned red Micro-QLED. **d**, pixel-sizes-dependent peak EQEs of multiple QLED devices (pristine red QLED and red Micro-QLED). Error bar represents the standard deviation of the data collected from > 6 samples.


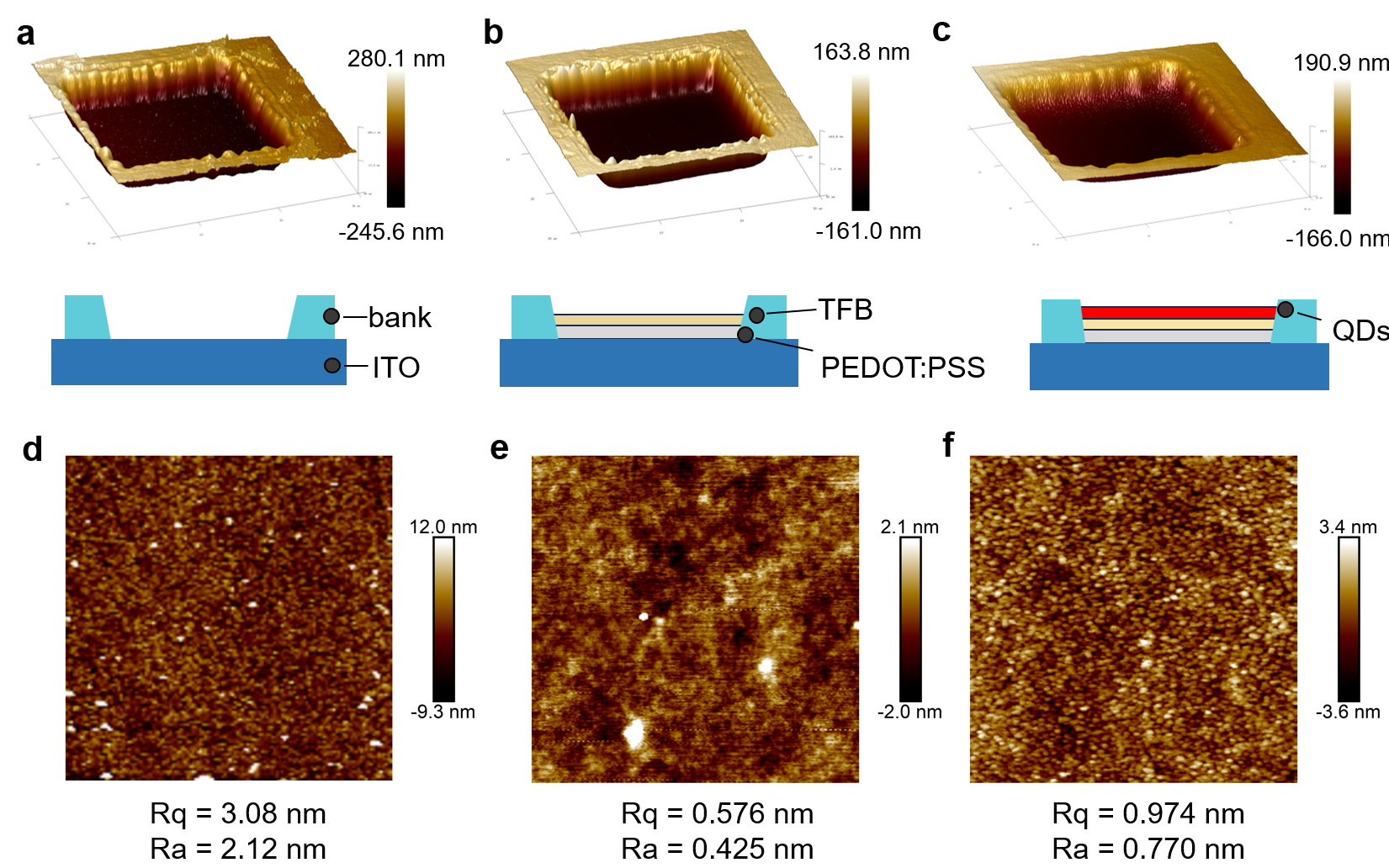


**Supplementary Fig. S10** **a–c**, AFM images in the Micro-QLED manufacturing process, including the 20 μm × 20 μm photolithography template, after preparing PEDOT: PSS (hole injection layer, HIL) and TFB layers, and after preparing QDs layers. **d–f** is a magnified view of the selected square within the pixel area.


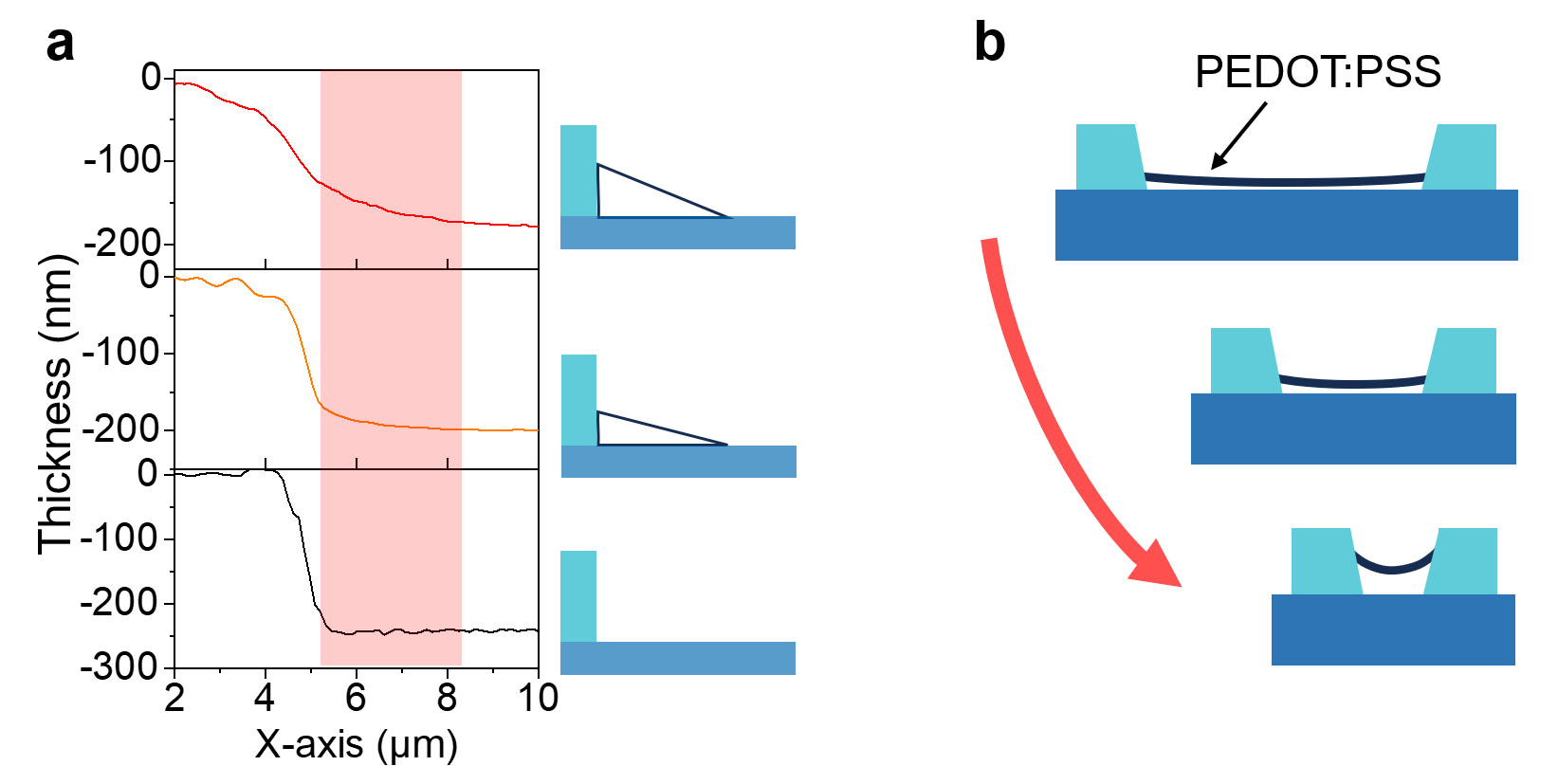


**Supplementary Fig. S11 a,** Height profiles of atomic force microscopy (AFM) images in the Micro-QLED manufacturing process. **b,** Schematics diagram of PEDOT: PSS film based on reduced pixels size.


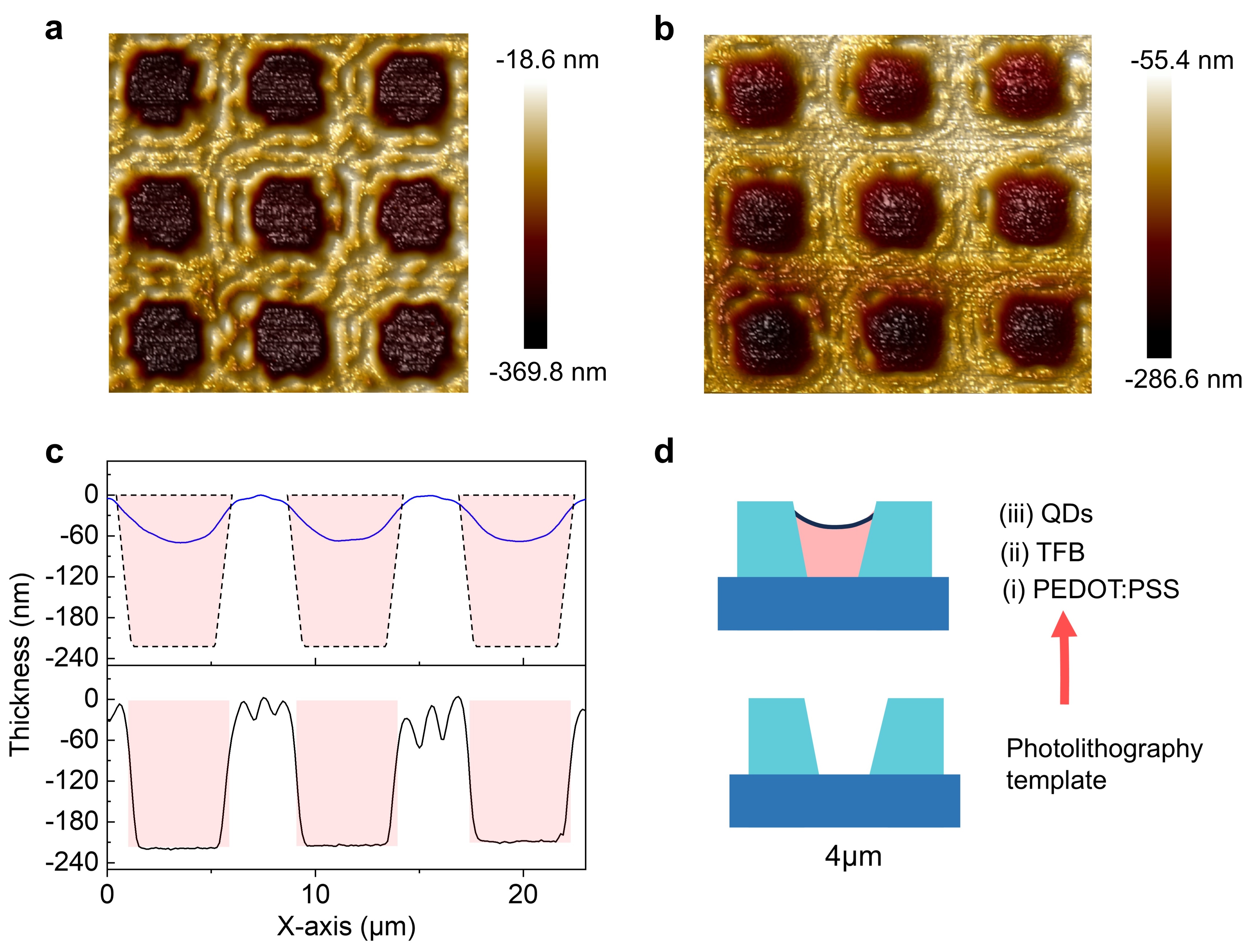


**Supplementary Fig. S12 a,** AFM image of the 4μm × 4μm photolithography template. **b**, AFM image of Micro-QLED device with PEDOT: PSS, TFB and QDs layers. **c**, Height profiles of AFM images. **d,** Schematics diagram of multi-functional films based on the photolithography template with 4 μm × 4 μm pixels size.


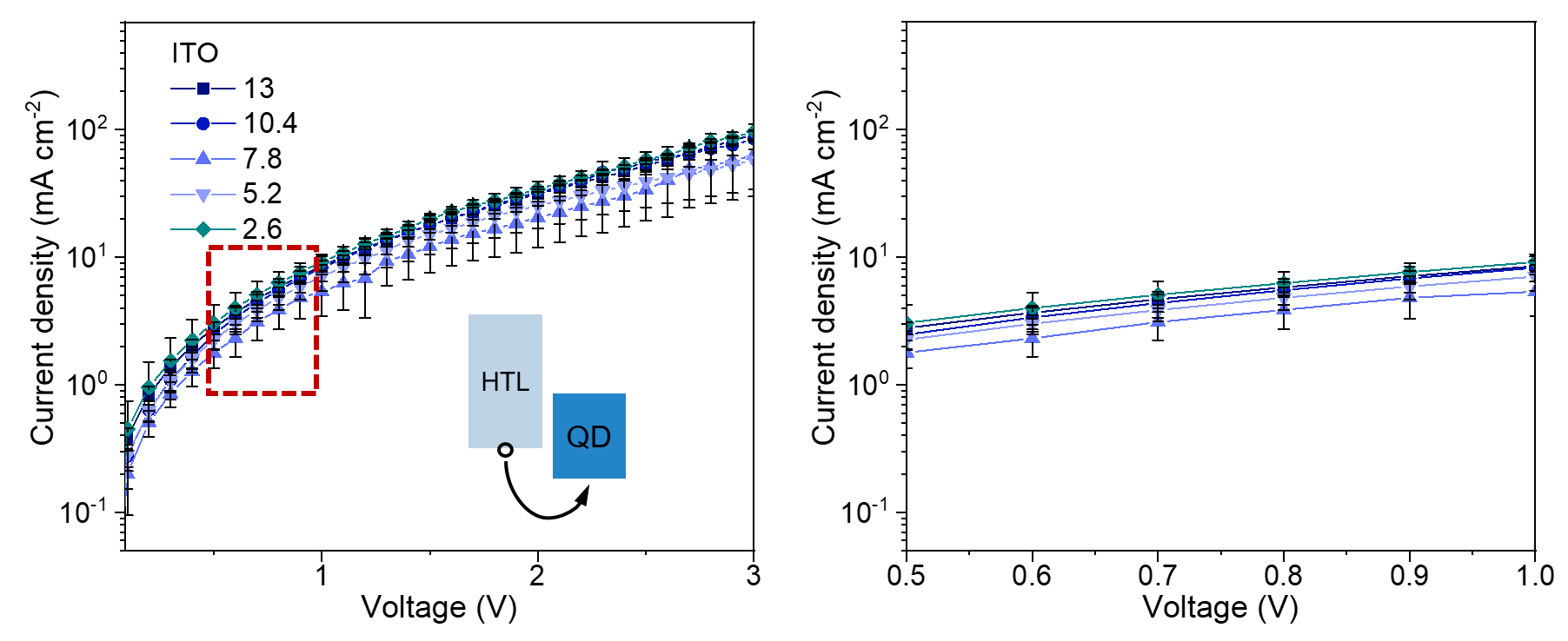


**Supplementary Fig. S13** Current density-voltage characteristics of hole-only device using the pristine ITO substrate (reduced HIL concentration). Hole-only device structure: PEDOT: PSS/TFB/QDs/MoO_3_/Al. The right image is an enlarged version of the left with the voltage range is 0.5 – 1 V.


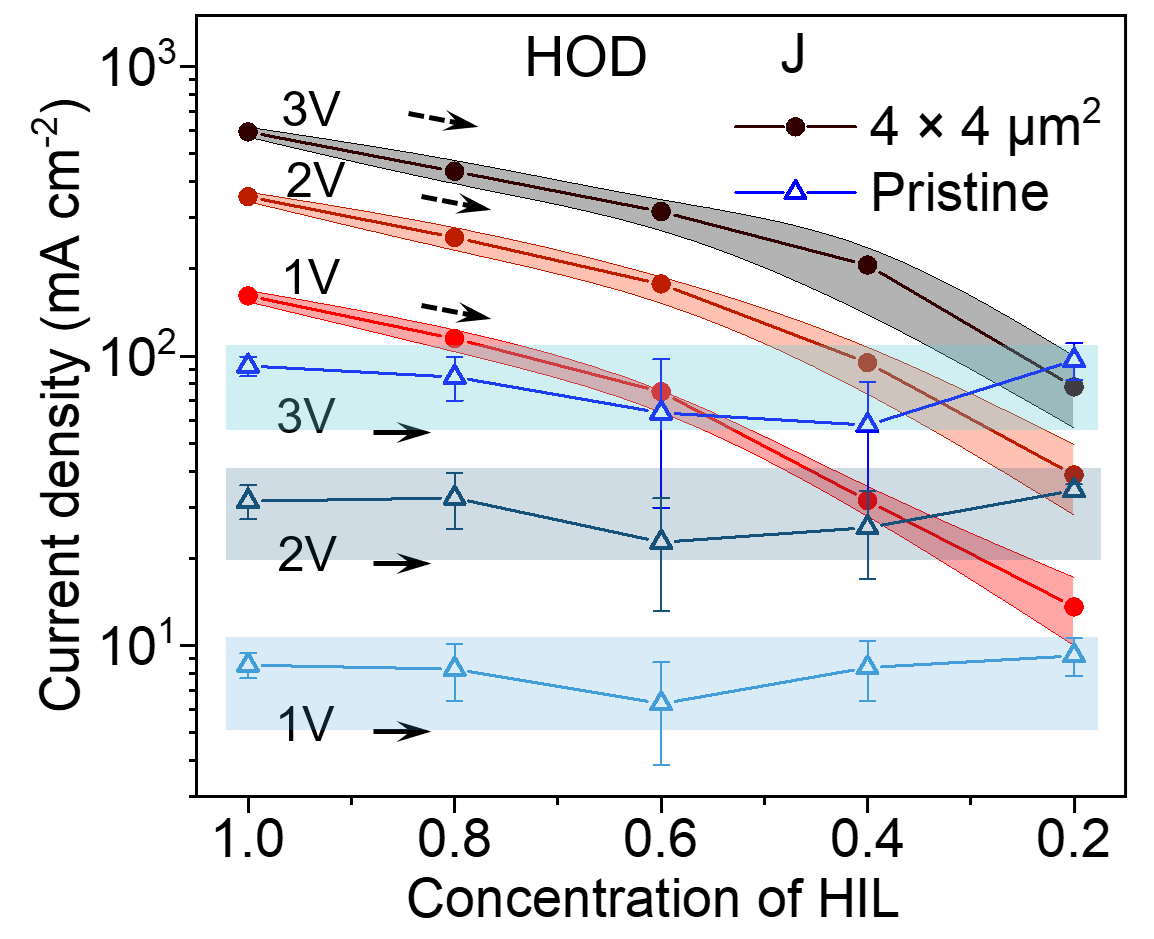


**Supplementary Fig. S14** HIL concentration-dependent current density characteristics of hole-only device.


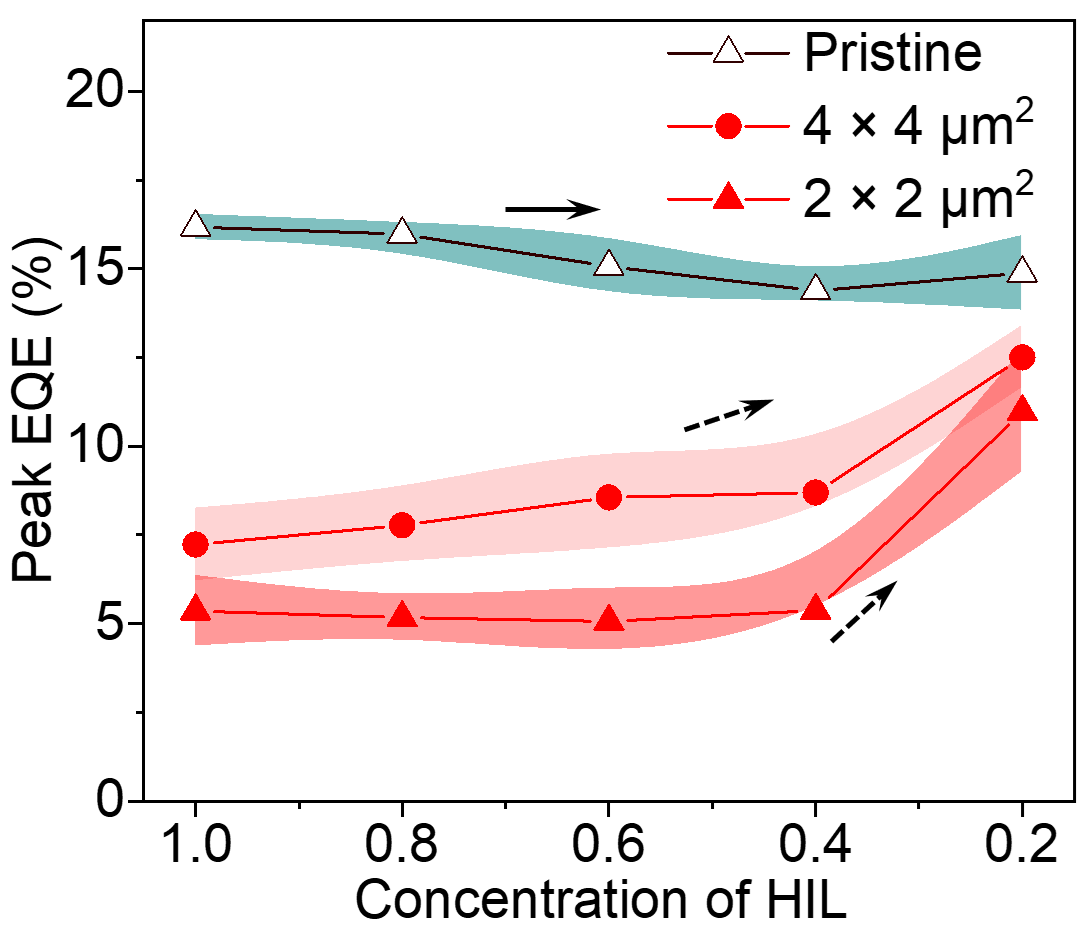


**Supplementary Fig. S15** Peak EQEs of red Micro-QLED versus HIL concentration, using the ITO substrate and the photolithography template. Error bar represents the standard deviation of the data collected from > 6 samples.


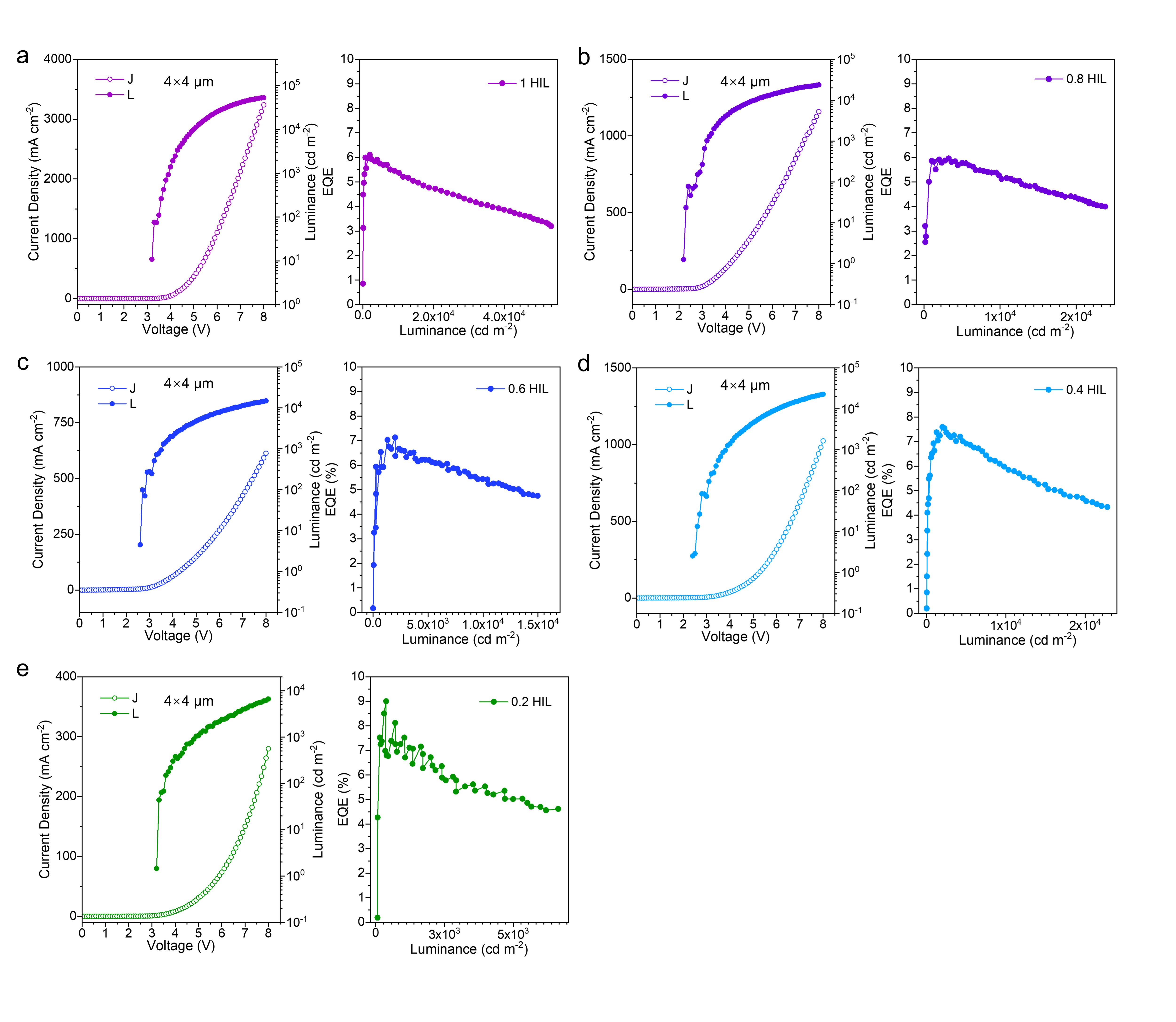


**Supplementary Fig. S16** J-V-L curves and EQE curves of blue Micro-QLED devices using the patterned template of 4 μm × 4 μm. The HIL concentration is 1 HIL (a), 0.8 HIL (b), 0.6 HIL (c), 0.4 (d), 0.2 HIL (e).


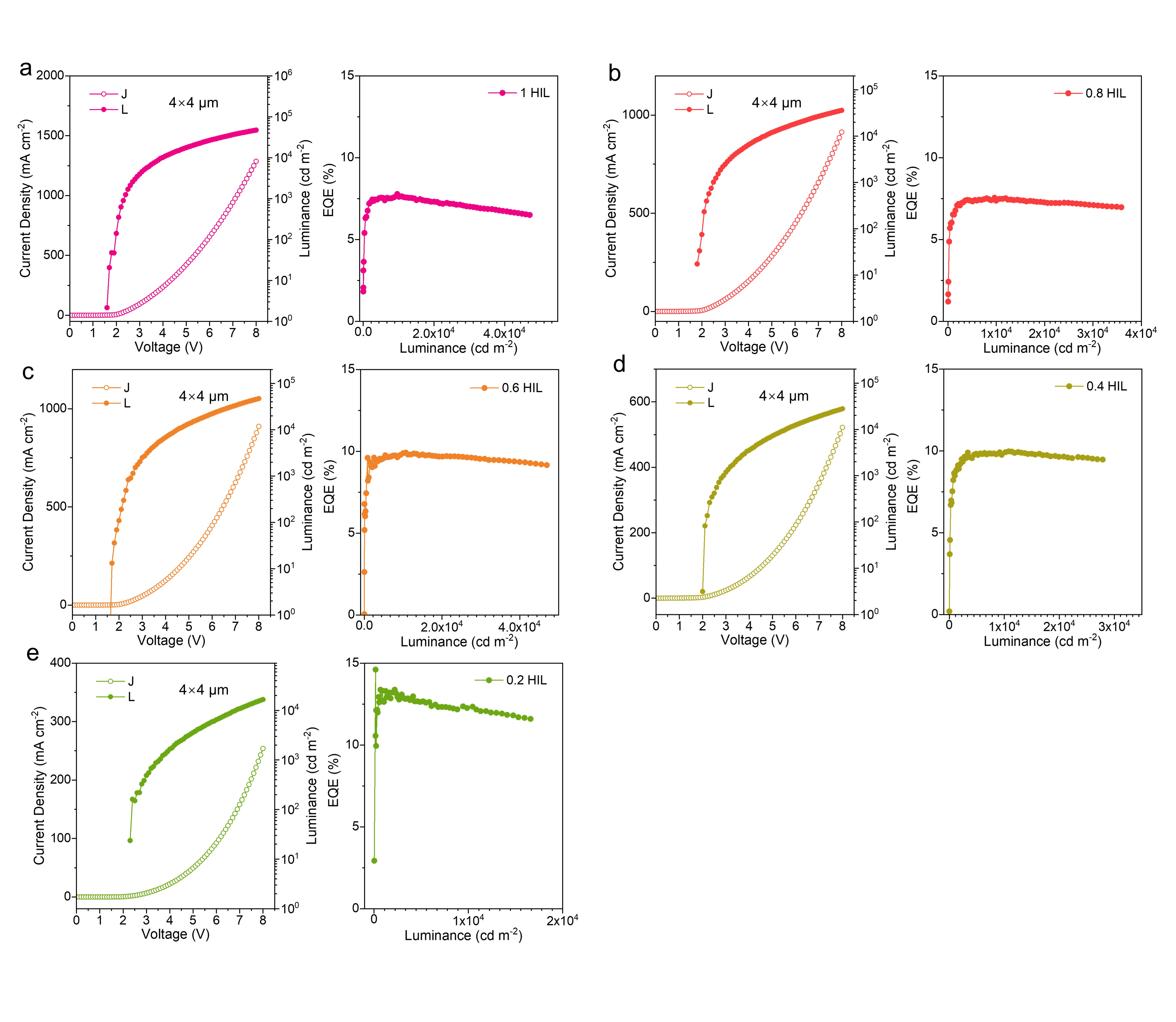


**Supplementary Fig. S17** J-V-L curves and EQE curves of red Micro-QLED devices using the patterned template of 4 μm × 4 μm. The HIL concentration is 1 HIL (a), 0.8 HIL (b), 0.6 HIL (c), 0.4 (d), 0.2 HIL (e).


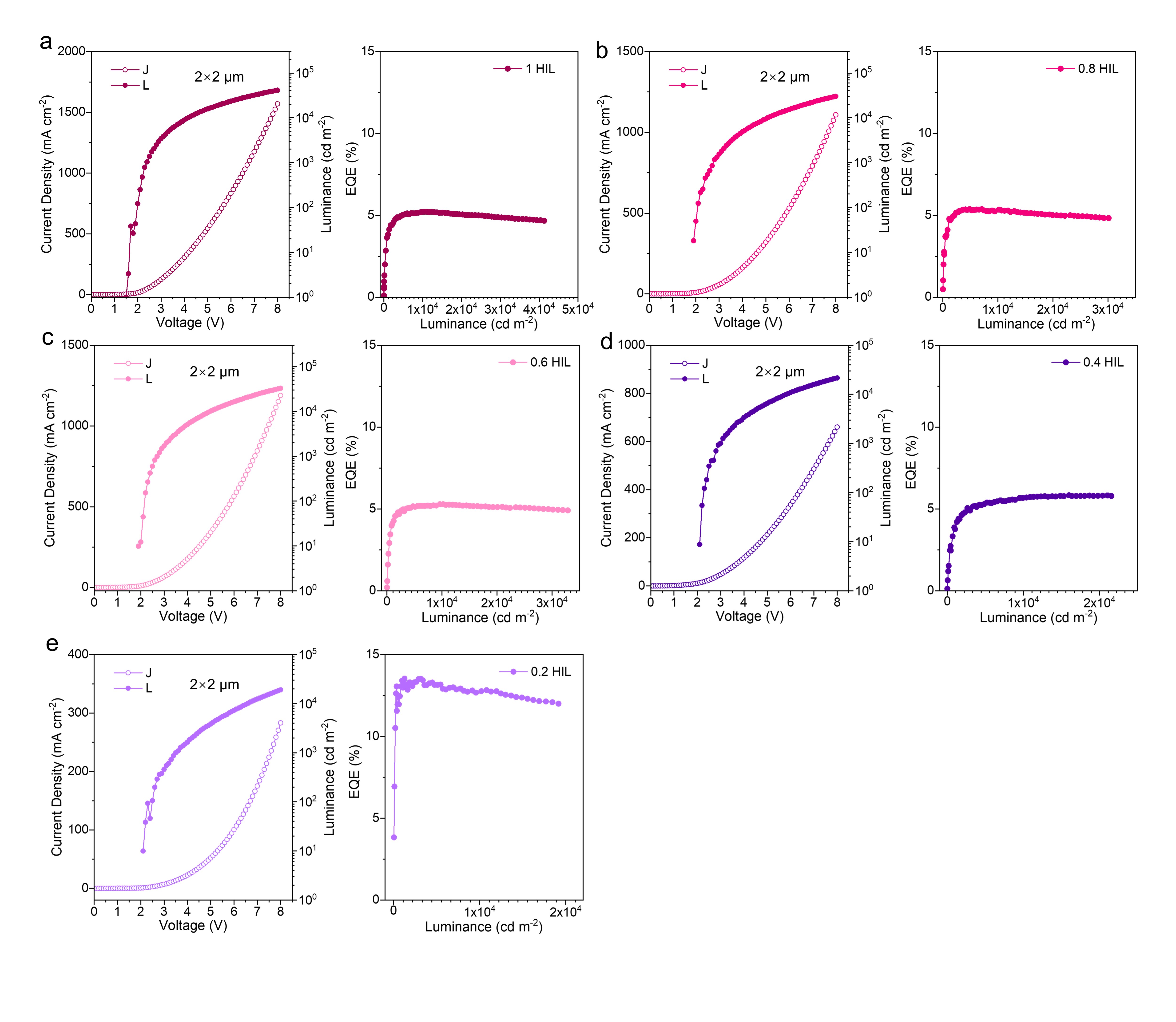


**Supplementary Fig. S18** J-V-L curves and EQE curves of red Micro-QLED devices using the patterned template of 2 μm × 2 μm. The HIL concentration is 1 HIL (a), 0.8 HIL (b), 0.6 HIL (c), 0.4 (d), 0.2 HIL (e).


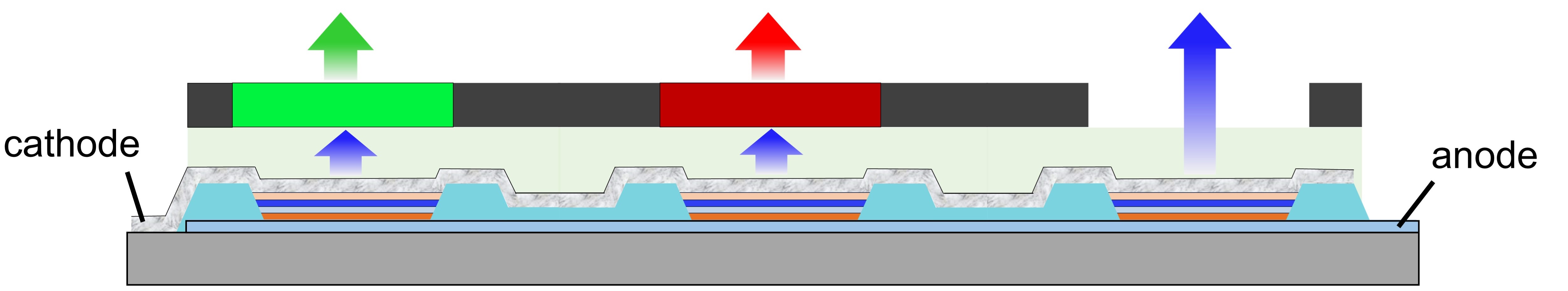


**Supplementary Fig. S19** The schematic diagram of full-color Micro-QLED pixel.


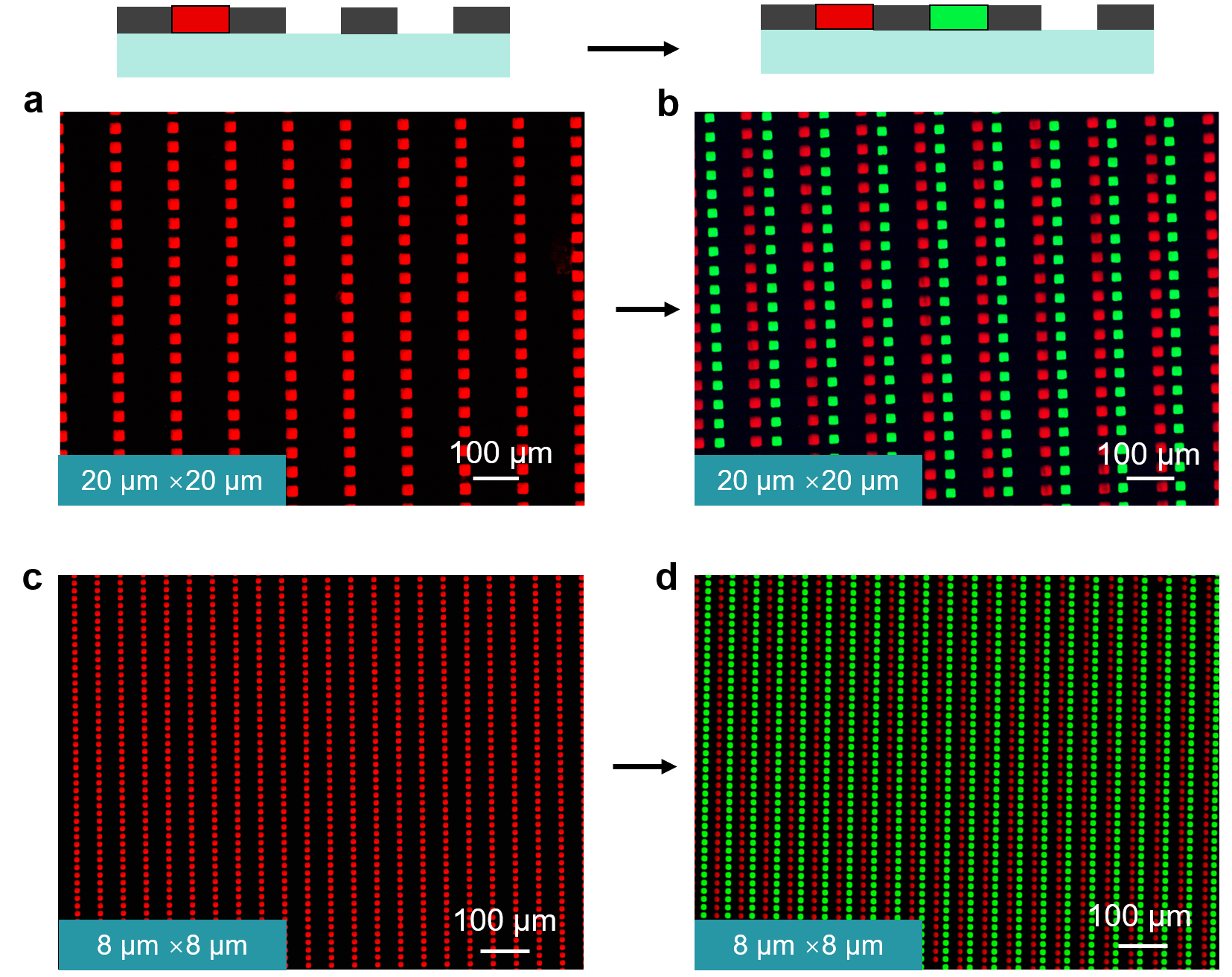


**Supplementary Fig. S20** QDCC fabrication process and optical microscope images with a pixel size of 20 μm × 20 μm (a, b) and 8 μm × 8 μm (c, d).


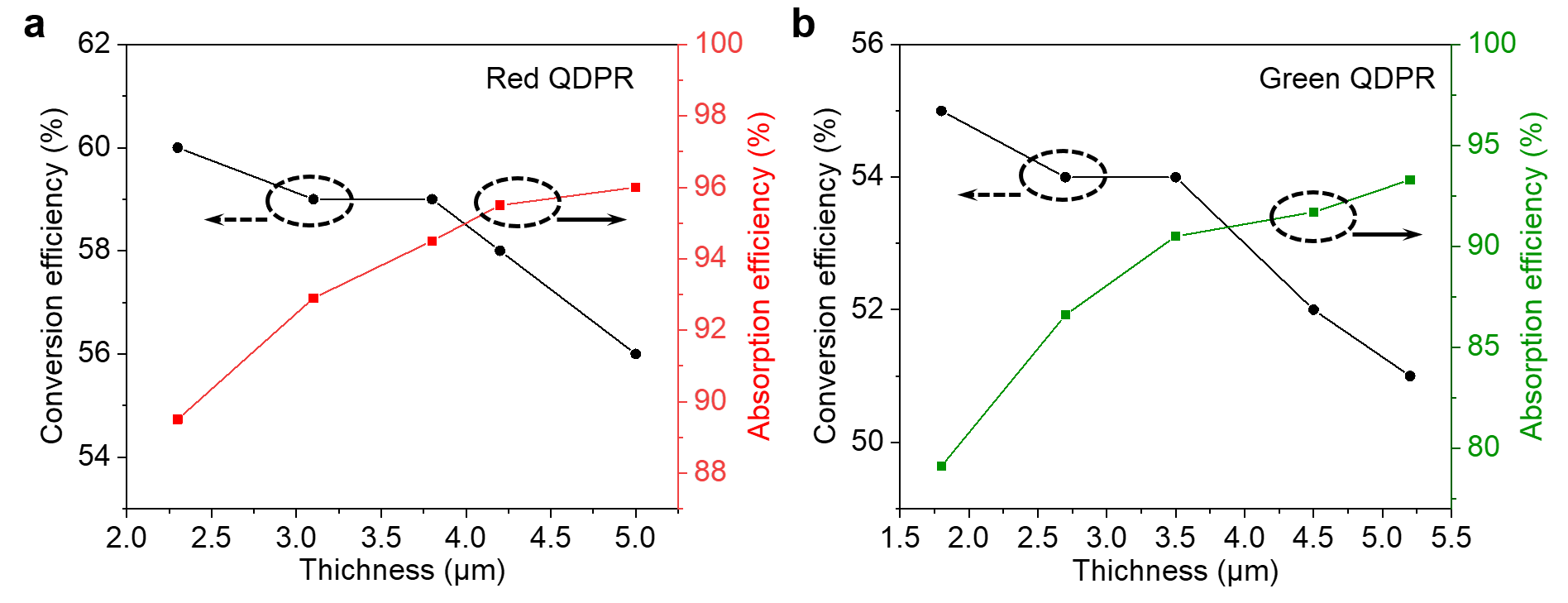


**Supplementary Fig. S21** The thickness dependent blue light absorbance efficiency and photoconversion efficiency for red and green QDPR films.


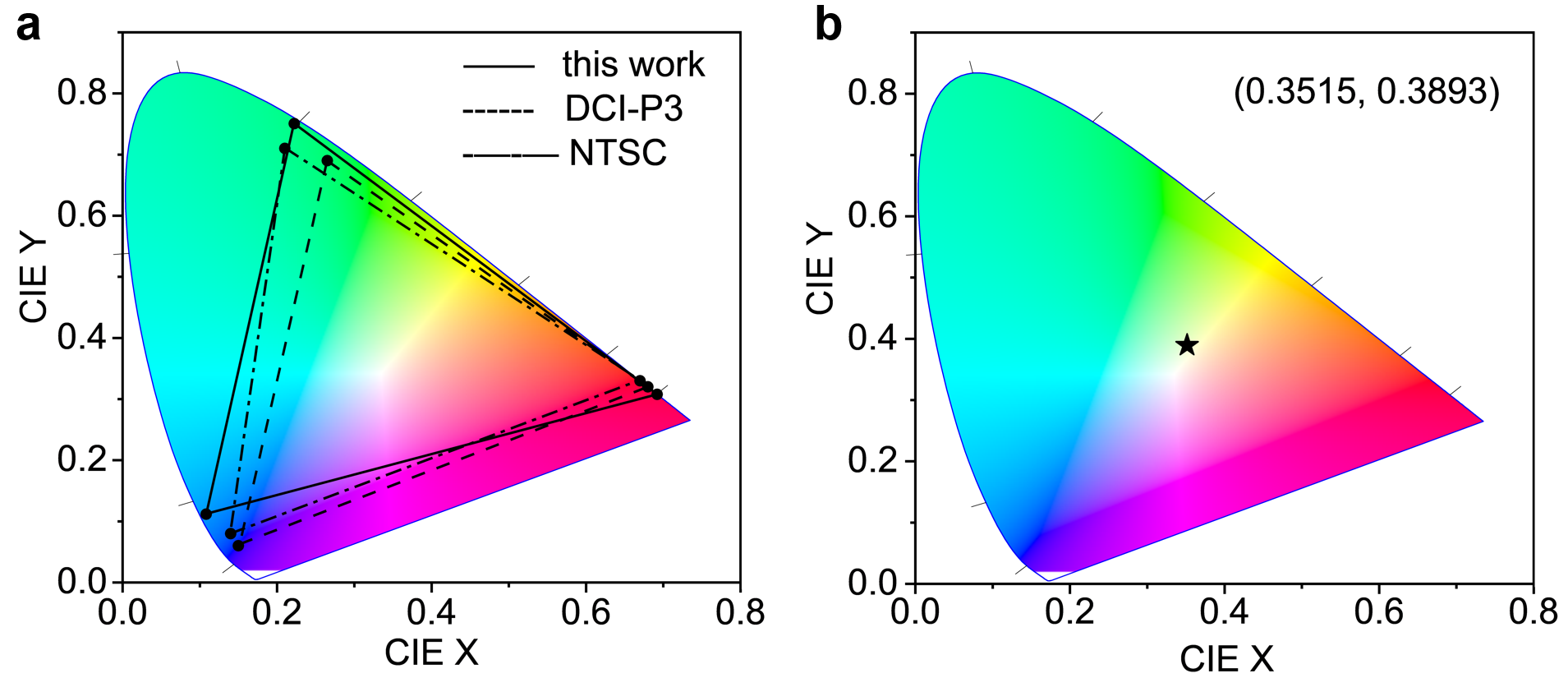


**Supplementary Fig. S22** **a,** Color gamut of the full-color color-converted Micro-QLED in the CIE color space established in 1931 (CIE 1931). The CIE coordinate of the blue light is (0.1082, 0.1118), while the CIE coordinates of the green and red emissions are (0.2223, 0.7505) and (0.6921, 0.3078). The area of the color-converted Micro-QLEDs was 110.8% of the NTSC color gamut with a coverage ratio of 95.8%, 115.3% of the DCI-P3 color gamut with a coverage ratio of 91.1%. **b,** CIE coordinates of the color-converted device. The CIE coordinate of the white light is (0.3515, 0.3893).


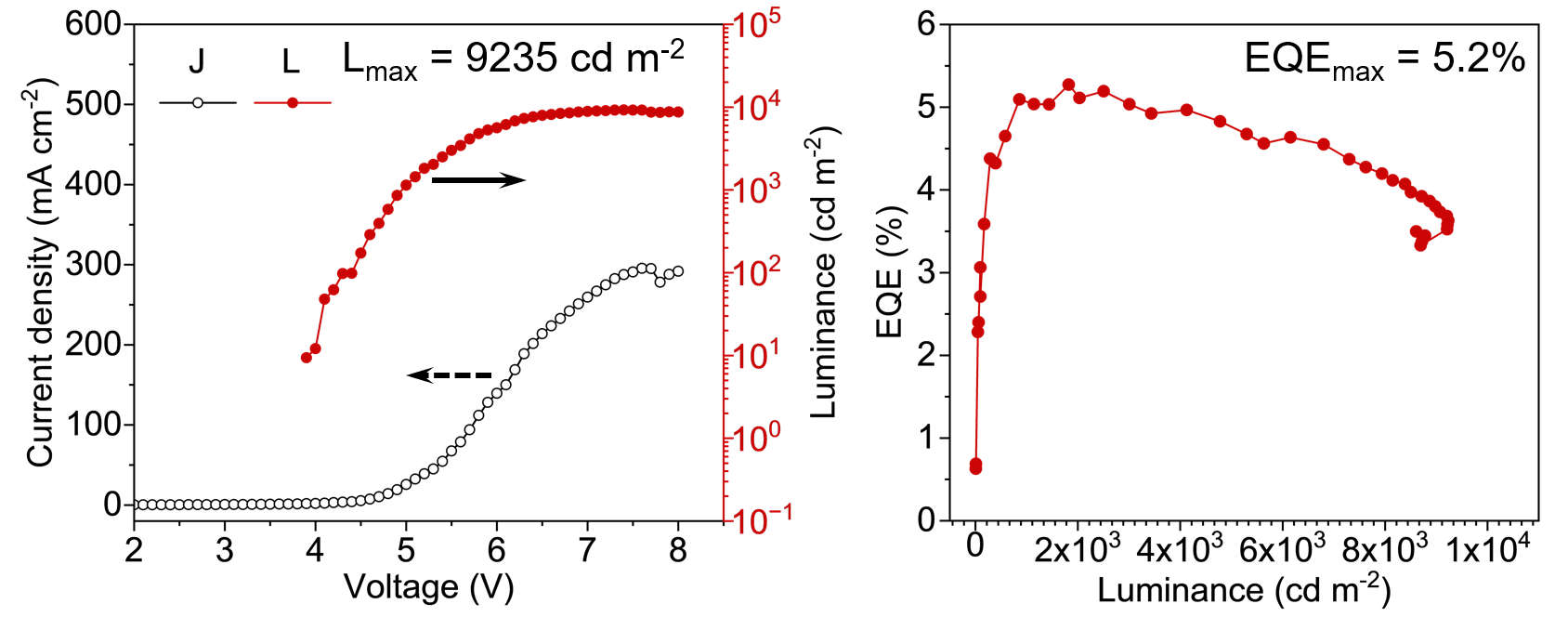


**Supplementary Fig. S23** J- V- L curves and EQE curves versus luminance of color-converted Micro-QLED with QDCC of 20 μm × 20 μm.


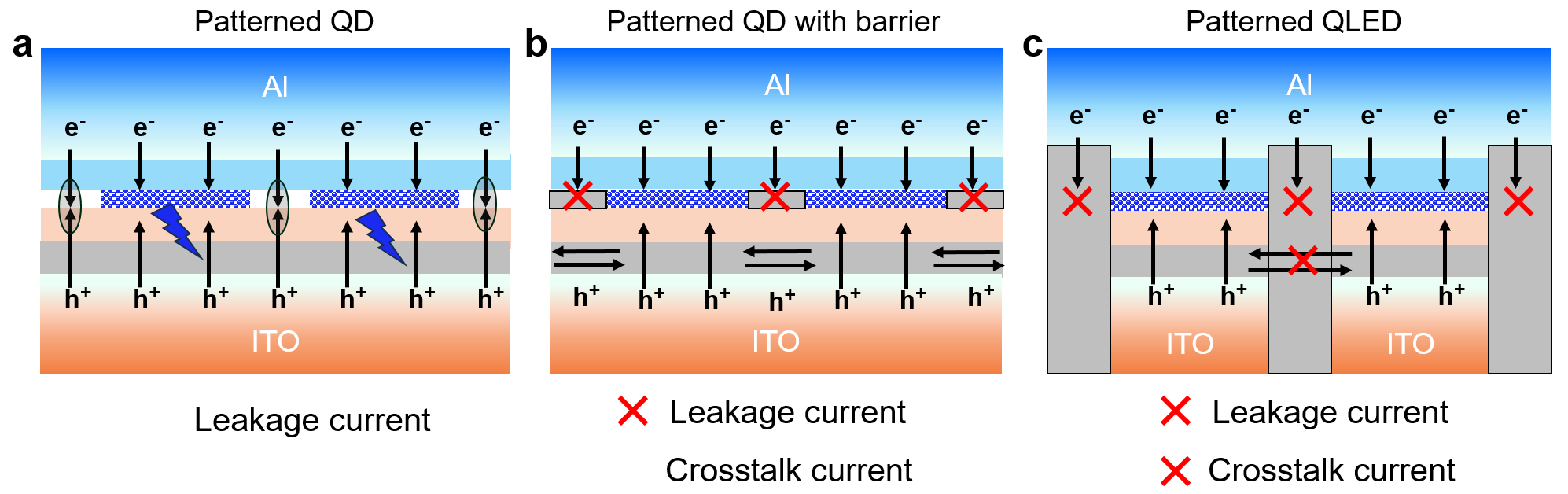


**Supplementary Fig. S24** The schematic diagram of a single Micro-QLED pixel with electron and hole injection.


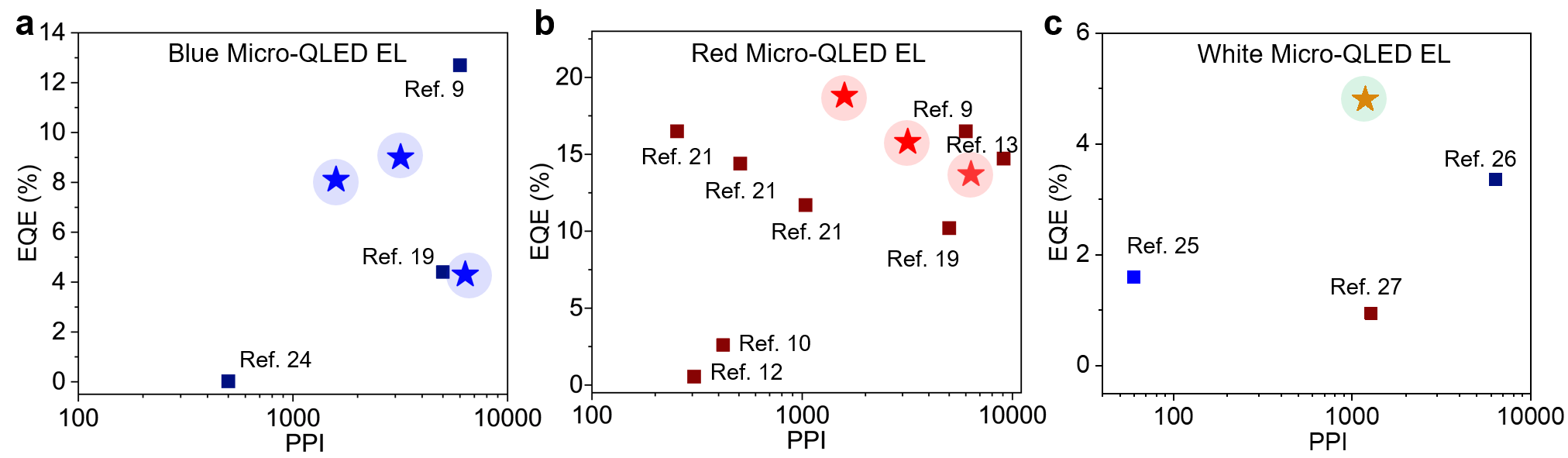


**Supplementary Fig. S25** Comparison of Micro-QLED performance including EQE and PPI (patterned Micro-QLED EL) in this work (five-pointed star) and other reported works. Note: reference 9 was published during the revision of this work.

**References**

1. Ko J. et al. Direct Photolithographic Patterning of Colloidal Quantum Dots Enabled by UV-Crosslinkable and Hole-Transporting Polymer Ligands. *ACS Appl. Mater. Interfaces* **12,** 42153-42160 (2020).

2. Cho H. C. et al. Direct Optical Patterning of Quantum Dot Light-Emitting Diodes via In Situ Ligand Exchange. *Adv. Mater.* **32,** e2003805 (2020).

3. Yang J. et al. High-resolution patterning of colloidal quantum dots via non-destructive, light-driven ligand crosslinking. *Nat. Commun.* **11,** 2874 (2020).

4. Wang Y. et al. Direct Optical Patterning of Nanocrystal-Based Thin-Film Transistors and Light-Emitting Diodes through Native Ligand Cleavage. *ACS Appl. Nano Mater.* **5,** 8457-8466 (2022).

5. Yang J. et al. Nondestructive Photopatterning of Heavy-Metal-Free Quantum Dots. *Adv. Mater.* **34,** e2205504 (2022).

6. Lu S. Y. et al. Beyond a Linker: The Role of Photochemistry of Crosslinkers in the Direct Optical Patterning of Colloidal Nanocrystals. *Angew. Chem. Int. Ed.* **61,** e202202633 (2022).

7. Hahm D. et al. Direct patterning of colloidal quantum dots with adaptable dual-ligand surface. *Nat. Nanotechnol.* **17,** 952-958 (2022).

8. Liu D. et al. Direct optical patterning of perovskite nanocrystals with ligand cross-linkers. *Science Advances* **8,** eabm8433 (2022).

9. Wang C. Y. et al. High-resolution and high-efficiency micro quantum-dot light-emitting diode arrays via conventional photolithography. *Nano Res.* (2025).

10. Kim B. H. et al. High-resolution patterns of quantum dots formed by electrohydrodynamic jet printing for light-emitting diodes. *Nano Lett.* **15,** 969-973 (2015).

11. Wang H. W. et al. High-efficiency and high-resolution patterned quantum dot light emitting diodes by electrohydrodynamic printing. *Nanoscale Adv.* **5,** 1183-1189 (2023).

12. Li H. G. et al. High‐Resolution Pixelated Light Emitting Diodes Based on Electrohydrodynamic Printing and Coffee‐Ring‐Free Quantum Dot Film. *Adv. Mater. Technol.* **5,** 2000401 (2020).

13. Meng T. T. et al. Ultrahigh-resolution quantum-dot light-emitting diodes. *Nat. Photonics* **16,** 297-303 (2022).

14. Wang K. et al. Wettability-Guided Screen Printing of Perovskite Microlaser Arrays for Current-Driven Displays. *Adv. Mater.* **32,** e2001999 (2020).

15. Kwon J. I. et al. Ultrahigh-resolution full-color perovskite nanocrystal patterning for ultrathin skin-attachable displays. *Science Advances* **8,** eadd0697 (2022).

16. Yoo J. et al. Highly efficient printed quantum dot light-emitting diodes through ultrahigh-definition double-layer transfer printing. *Nat. Photonics* **18,** 1105-1112 (2024).

17. Zhao J. Y. et al. Large-area patterning of full-color quantum dot arrays beyond 1000 pixels per inch by selective electrophoretic deposition. *Nat. Commun.* **12,** 4603 (2021).

18. Luo C. Z. et al. High‐Resolution, Highly Transparent, and Efficient Quantum Dot Light‐Emitting Diodes. *Adv. Mater.* **35,** (2023).

19. Ma T. et al. One‐Step, Mask‐Free, Rapid Laser Writing Fabrication of Electroluminescent Perovskite@Oxide Pixels for Ultra‐High PPI, Efficient Micro‐QLEDs. *Adv. Funct. Mater.* **35,** 2413811 (2025).

20. Liang S. Y. et al. High‐Resolution Patterning of 2D Perovskite Films through Femtosecond Laser Direct Writing. *Adv. Funct. Mater.* **32,** (2022).

21. Luo C. Z. et al. Ultrahigh-resolution, high-fidelity quantum dot pixels patterned by dielectric electrophoretic deposition. *Light Sci. Appl.* **13,** 273 (2024).

22. Kim T.-H. et al. Full-colour quantum dot displays fabricated by transfer printing. *Nat. Photonics* **5,** 176-182 (2011).

23. Li Y. Z. et al. 80‐1: Invited Paper: Developing AMQLED Technology for Display Applications. *SID. Symp. Dig. Tech. Pap.* **49,** 1076-1079 (2018).

24. Mei W. H. et al. High-resolution, full-color quantum dot light-emitting diode display fabricated via photolithography approach. *Nano Res.* **13,** 2485-2491 (2020).

25. Choi M. K. et al. Wearable red–green–blue quantum dot light-emitting diode array using high-resolution intaglio transfer printing. *Nat. Commun.* **6,** 7149 (2015).

26. Yang K. Y. et al. High-resolution and high-performance full-color electroluminescent quantum dot light-emitting diodes. *Nano Energy* 110817 (2025).

27. Li X. X. et al. CdSe/ZnS Quantum Dot Patterned Arrays for Full-Color Light-Emitting Diodes in Active-Matrix QLED Display. *ACS Appl. Nano Mater.* **7,** 9086-9094 (2024).
